# A comprehensive analysis of lung cancer highlighting epidemiological factors and psychiatric comorbidities from the All of Us Research Program

**DOI:** 10.1038/s41598-023-37585-0

**Published:** 2023-07-05

**Authors:** Vikram R. Shaw, Jinyoung Byun, Rowland W. Pettit, Younghun Han, David A. Hsiou, Luke A. Nordstrom, Christopher I. Amos

**Affiliations:** 1 Institute for Clinical and Translational Research, Baylor College of Medicine, One Baylor Plaza, Houston, TX 77030 USA; 2 Section of Epidemiology and Population Sciences, Department of Medicine, Baylor College of Medicine, Houston, TX USA; 3 Dan L Duncan Comprehensive Cancer Center, Baylor College of Medicine, Houston, TX USA; 4 School of Medicine, Baylor College of Medicine, Houston, TX USA

**Keywords:** Cancer epidemiology, Lung cancer, Risk factors

## Abstract

Lung cancer is the leading cause of cancer-related mortality in the United States. Investigating epidemiological and clinical parameters can contribute to an improved understanding of disease development and management. In this cross-sectional, case–control study, we used the All of Us database to compare healthcare access, family history, smoking-related behaviors, and psychiatric comorbidities in light smoking controls, matched smoking controls, and primary and secondary lung cancer patients. We found a decreased odds of primary lung cancer patients versus matched smoking controls reporting inability to afford follow-up or specialist care. Additionally, we found a significantly increased odds of secondary lung cancer patients having comorbid anxiety and insomnia when compared to matched smoking controls. Our study provides a profile of the psychiatric disease burden in lung cancer patients and reports key epidemiological factors in patients with primary and secondary lung cancer. By using two controls, we were able to separate smoking behavior from lung cancer and identify factors that were mediated by heavy smoking alone or by both smoking and lung cancer.

## Introduction

In 2022, approximately 236,000 lung cancer diagnoses and 130,000 lung cancer deaths were expected to occur in the United States (US)^1^. Every day, roughly 350 patients are expected to die from lung cancer, making it the leading cause of cancer-related death in the US^1^. Tumors in the lungs can be classified as primary lung cancer, including small-cell or non-small-cell lung cancer, or secondary lung cancer, which typically arises from the metastasis of breast^2^, colorectal^3^, renal^4^, testicular^5^, and uterine cancer^6^, among other forms of cancer. Many primary lung cancers are attributed to modifiable risk factors, such as smoking^1,7^, secondhand smoke^7^, excess body weight^8^, red and processed meat consumption^7^, alcohol intake^7^, and various occupational exposures^9^. However, cigarette smoking is a well-known risk factor for primary lung cancer and is attributed as the leading cause of more than 80% of lung cancer cases in the US^1^.

Although cigarette smoking is a significant risk factor for the development of lung cancer, numerous studies have demonstrated that a family history of lung cancer is also associated with an increased risk^10^. Even after accounting for age, sex, smoking history, and occupation, studies suggest a 2–4-fold increase in lung cancer risk for first-degree relatives of lung cancer patients^10^. Other epidemiologic factors, such as barriers to healthcare, can impact lung cancer development and outcomes, especially in vulnerable populations^11^. Studies estimate that only 5–18% of patients at high risk for lung cancer receive low dose computed tomography (LDCT) screening^12^. Investigating smoking-related behaviors is also crucial in the context of lung cancer risks, including e-cigarette use and smokeless tobacco. While nicotine replacement and pharmacological therapies along with behavior therapies have led to improved smoking cessation rates, the accessibility of e-cigarettes has led to an increase in their usage^13,14^. A particular concern is that e-cigarette users often also use cigarettes, thus increasing their lung cancer risk^15^. Notably, a literature gap exists in understanding the complex interplay between smoking, e-cigarette or smokeless tobacco use, and lung cancer, which this study aims to address.

Finally, the psychiatric disease burden associated with both smoking and lung cancer is well-documented^16–18^. However, to our knowledge, no studies have investigated the differences in the psychiatric disease burden between primary and secondary lung cancer. Understanding which psychiatric diseases are comorbid with both primary and secondary lung cancer can help physicians develop treatment plans tailored to the individual patient.

To obtain a more comprehensive understanding of lung cancer development, treatment, and outcomes, it is essential to investigate epidemiological factors beyond cigarette smoking. This investigation can help develop better risk-based lung cancer screening methods and outcome prediction models that can draw on data from diverse sources^19^. This study aims to explore several key factors that may contribute to primary and secondary lung cancer, including lung cancer family history, barriers to healthcare, smoking-related behaviors, and psychiatric comorbidities. To understand better the impact of these factors, we designed a case–control analysis using two control groups (light smokers and matched smokers) to study the effects of smoking, lung cancer, and comorbid psychiatric conditions. Specifically, this study aims to answer the research question of whether the prevalence and impact of smoking-related behaviors, psychiatric comorbidities, and other epidemiological factors differ between primary and secondary lung cancer patients compared to light smoking and matched smoking controls.

## Materials and methods

### All of Us Research Program

The All of Us Research Program is a prospective cohort study with the objective of recruiting at least one million individuals in the US to provide a comprehensive database that enables researchers to investigate the effects of lifestyle, access to care, family history, environment, and genomics on participant health^20^. The program collects data through self-reported surveys, electronic health records (EHRs), and physical wearables such as Fitbit devices. Of the 372,082 patients in the All of Us Research Program, 54.1% are white, 19.7% are black or African American, 3.3% are Asian, 0.60% are Middle Eastern or North African, and 0.11% are Native Hawaiian or Other Pacific Islander. Data from this program are accessible at http://www.allofus.nih.gov, and this study was conducted on version 6 of the data utilizing the All of Us Researcher Workbench. Supplementary Material provide codes utilized to query EHRs for lung cancer and psychiatric conditions.

### Lung cancer patient and control selection

Using the cohort builder function within the All of Us workbench, we created cohorts for patients with primary and secondary lung cancer based on source concept names (Supplementary Material). To protect individual-level patient information and in accordance with the All of Us data access policy, we excluded a small number of patients from both the primary and secondary lung cancer cohorts whose sex at birth survey answer categories contained fewer than 20 participants. Controls were divided into two groups: a light smoking control (LSC) and a matched smoking control (MSC). Light smoking controls in primary lung cancer and secondary lung cancer are designated as LSC-1 and LSC-2, respectively. Matched smoking controls in primary and secondary lung cancer are designated as MSC-1 and MSC-2, respectively. Control group participants were matched with patients based on their current age at the time of this study in 5-year intervals, sex at birth, and smoking status from a sample excluding primary and secondary lung cancer patients. The controls were matched by randomly selecting the control group participant with the appropriate inclusion criteria for a given matched lung cancer patient from a list of eligible control participants (i.e., same age, sex at birth, and smoking status as matched lung cancer patient). While smoking pack years is a well-established metric for smoking history^21^, we used the number of years smoked as the matching criteria because not all patients filled out both years smoked and the average number of daily cigarettes, which are needed to calculate the pack-year metric. LSC controls answered the “Number of Years Smoked” question from the “Lifestyle” survey with an answer less than or equal to 5, which is a well-published “years smoked” cutoff for light smokers^22,23^, while MSC controls were matched based on the exact number of years smoked. Fewer secondary lung cancer patients completed the “Number of Years Smoked” question, leading to a smaller sample size for the matched smoking controls in secondary lung cancer. We excluded answers of “PMI: Skip” and “PMI: Don’t Know” when calculating smoking-related demographic information such as the average daily cigarette number, the current average daily cigarette number, the daily smoking starting age, and the number of years smoked.

### Statistical analysis

Odds ratios were used to generate forest plots, and the following R (v 4.2.2) packages were used for statistical analysis or plotting: epitools (v 0.5.10.1)^24^, tidyverse (v 1.3.2)^25^, patchwork (v 1.1.2)^26^, and ggplot2 (v 3.4.0)^27^. Mid p-values are commonly used in the analysis of odds ratios and are calculated by taking the midpoint of the range of p-values with a full description available in the documentation for the epitools^24^ R package. The epitools R package provides mid p-values, Fisher p-values, and Chi-squared p-values. Mid p-values are used for all p-values in this study except for the primary lung cancer vs. LSC and MSC vs. LSC comparisons for electronic cigarette use and in analysis of psychiatric comorbidities, in which cases Fisher exact p-values were used as the epitools program returned a value of 0 for the mid p-value. Bonferroni p-values were calculated by multiplying the shown p-value by the number of comparisons and are significant if they are less than 0.05. All p-values reported in results text are mid p-values.

## Results

### Lung cancer patient and control demographics

We conducted a matched case–control study to investigate the epidemiological and clinical parameters of primary and secondary lung cancer. This study included two age- and sex-matched controls for each case: a light smoking control (LSC) and a matched smoking control (MSC), with the latter having smoked for an equivalent number of years as their respective lung cancer patient. From a total of 221,125 patients in the All of Us database with available electronic health record (EHR) data, we identified 1451 patients with primary lung cancer (prevalence of 0.66%) and 1161 patients with secondary lung cancer (prevalence of 0.53%). The median age of lung cancer patients in our cohorts at the time of this study was 72 for primary lung cancer and 67 for secondary lung cancer (Table 1), which aligns with the literature suggesting a median age of lung cancer diagnosis 70 for both men and women^28^. In our primary lung cancer cohort, 60.0% of patients reported female sex at birth, while 55.1% of secondary lung cancer patients did so. In our primary lung cancer cohort, 68.8% of patients were white, 16.7% were black or African American, 2.4% were Asian, and 7.7% were Hispanic. In our secondary lung cancer cohort, 68.6% of patients were white, 10.1% were black of African American, 3.2% were Asian, and 14.8% were Hispanic.

**Table 1:** Demographic and smoking behavior for patients with primary or secondary lung cancer and controls.

	Primary lung cancer (n = 1451)	Light smoking control without primary lung cancer (n = 1433) (LSC-1)	Matched smoking control without primary lung cancer (n = 1051) (MSC-1)	Secondary lung cancer (n = 1161)	Light smoking control without secondary lung cancer (n = 1127) (LSC-2)	Matched smoking control without secondary lung cancer (n = 541) (MSC-2)
Current median age (IQR)	72 (65–78)	71 (64–78)	72 (66–78)	67 (58–75)	68 (59–75)	70 (61–76)
Sex at birth, female	871 (60.0)	859 (59.9)	616 (58.6)	640 (55.1)	623 (55.3)	270 (49.9)
Race						
White	999 (68.8)	1142 (79.7)	660 (62.8)	797 (68.6)	839 (74.4)	344 (63.6)
Black or African American	243 (16.7)	114 (8.0)	237 (22.5)	117 (10.1)	120 (10.6)	123 (22.7)
Asian	35 (2.4)	NA (NA)	NA (NA)	37 (3.2)	NA (NA)	NA (NA)
Other	174 (12.0)	NA (NA)	NA (NA)	210 (18.1)	NA (NA)	NA (NA)
Ethnicity						
Hispanic or Latino	112 (7.7)	119 (8.3)	105 (10.0)	172 (14.8)	113 (10.0)	60 (11.1)
Not Hispanic or Latino	1288 (88.8)	1274 (88.9)	913 (86.9)	958 (82.5)	991 (87.9)	472 (87.2)
Other	51 (3.5)	40 (2.8)	33 (3.1)	31 (2.7)	23 (2.0)	NA (NA)
Income						
< $25k	357 (24.6)	194 (13.5)	316 (30.1)	222 (19.1)	177 (15.7)	172 (31.8)
$25–50k	252 (17.4)	213 (14.9)	177 (16.8)	152 (13.1)	146 (13.0)	80 (14.8)
$50–100k	241 (16.6)	355 (24.8)	206 (19.6)	243 (20.9)	292 (25.9)	91 (16.8)
$100k+	272 (18.7)	455 (31.8)	141 (13.4)	253 (21.8)	358 (31.8)	100 (18.5)
Not reported	329 (22.7)	216 (15.1)	211 (20.1)	291 (25.1)	154 (13.7)	98 (18.1)
Education						
Advanced degree	311 (21.4)	507 (35.4)	178 (16.9)	276 (23.8)	353 (31.3)	98 (18.1)
College graduate	286 (19.7)	386 (26.9)	200 (19.0)	284 (24.5)	317 (28.1)	93 (17.2)
Some college	410 (28.3)	318 (22.2)	297 (28.3)	285 (24.5)	278 (24.7)	151 (27.9)
High school graduate or GED	291 (20.1)	161 (11.2)	244 (23.2)	210 (18.1)	109 (9.7)	118 (21.8)
Other	153 (10.5)	61 (4.3)	132 (12.6)	106 (9.1)	70 (6.2)	81 (15.0)
Smoking						
100 cigarettes lifetime = yes	1057 (72.8)	1401 (97.8)	1037 (98.7)	541 (46.6)	1104 (98.0)	534 (98.7)
Daily cigarette median (IQR)	20 (10–25)	5 (3–10)	17 (10–20)	18 (10–20)	5 (3–10)	12 (5–20)
Current daily cigarette median (IQR)	0 (0–10)	0 (0–0)	0 (0–10)	0 (0–7)	0 (0–1)	0 (0–7)
Daily smoking starting age median (IQR)	16 (14–18)	18 (16.25–21)	17 (15–19)	17 (15–18)	18 (16–21)	17 (15–20)
Median number of years smoked (IQR)	35 (21.5–45)	3 (2–5)	35 (21–45)	25 (11–40)	3 (2–4)	24.5 (11–40)

Demographic and smoking behavior data is provided for primary and secondary lung cancer patients, as well as for light smoking (LSC) and matched smoking (MSC) controls. Parentheses indicate percentages except where noted as the interquartile range (IQR).

*NA is included if participant count < 20 to prevent patient identification and in accordance with *All of Us* policy.

The lifestyle survey data from participants offered insights into smoking behaviors and patterns. Of the primary lung cancer patients, 72.8% self-reported having smoked at least 100 cigarettes in their lifetime, compared to 46.6% of secondary lung cancer patients (Table 1). In the light smoking controls without primary lung cancer (LSC-1), the median years smoked was 3 (interquartile range [IQR]: 2–5). Primary lung cancer patients and matched smoking controls without primary lung cancer (MSC-1) reported a median of 35 years smoked (IQR: 21.5–45) and 35 years smoked (IQR: 21–45), respectively. In the light smoking controls without secondary lung cancer (LSC-2), the median years smoked was 3 (IQR: 2–4). Secondary lung cancer patients and matched smoking controls without secondary lung cancer (MSC-2) reported a median of 25 years smoked (IQR: 11–40) and 24.5 years smoked (IQR: 11–40), respectively.

### Differences in access to healthcare in primary and secondary lung cancer

**Figure 1:**
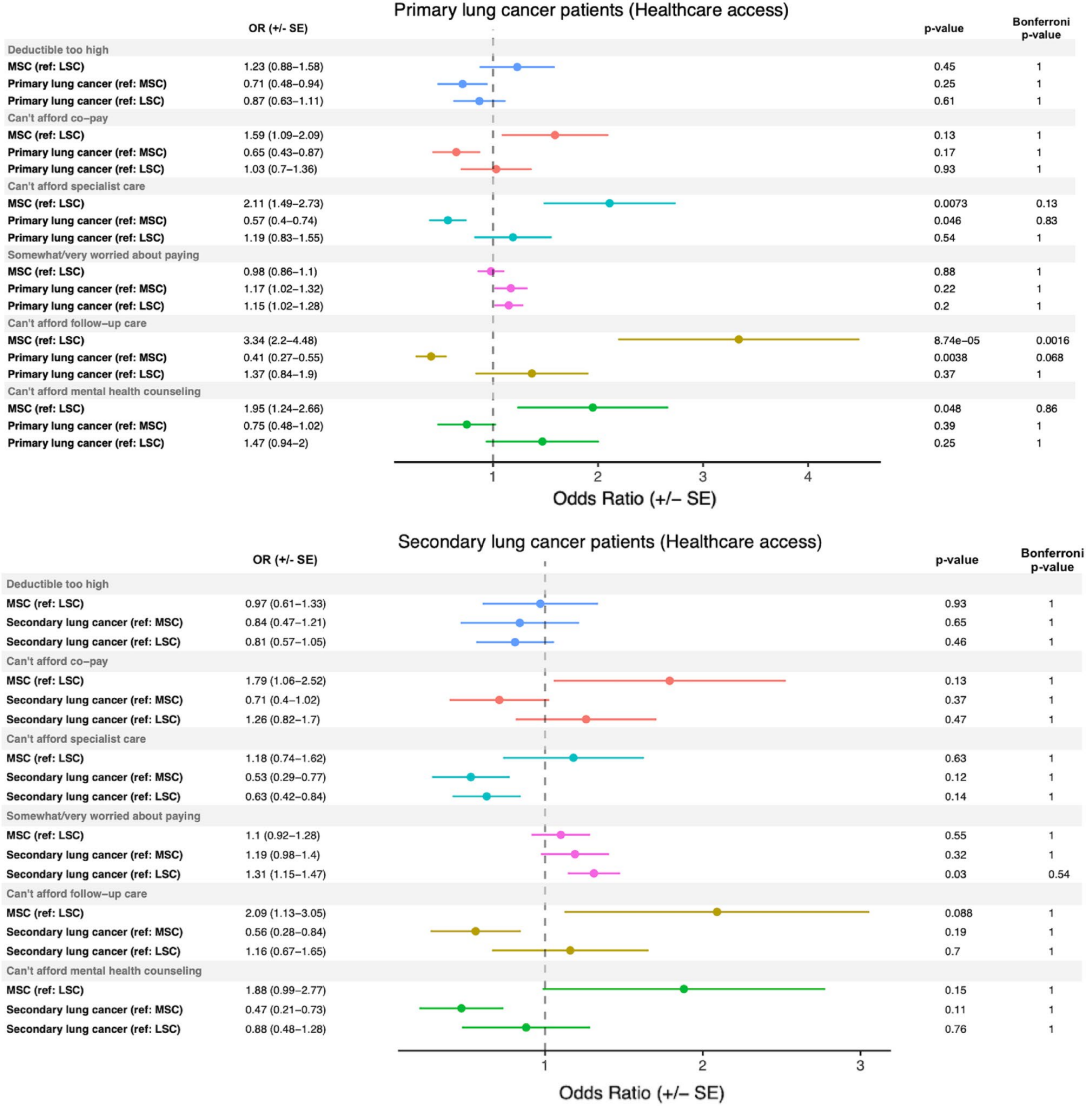
Healthcare access in primary and secondary lung cancer patients. Odds ratios (± standard error) generated comparing healthcare access patient-reported metrics in primary and secondary lung cancer patients to light smoking (LSC) and matched smoking (MSC) controls. The reference group (e.g., (ref: MSC)), mid-p value, and Bonferroni-corrected p-values are reported for each comparison.

After defining our cases and controls, we investigated several macro-level healthcare access factors, as well as patient-specific information such as smoking-related behavior and psychiatric comorbidities. We assessed the results from several healthcare access survey questions, including whether a patient could afford their co-pay, deductible, mental health counseling, or follow-up care, and whether they were worried about paying. The results of our analysis showed that primary lung cancer patients had significantly lower odds of reporting that they could not afford specialist or follow-up care, compared to MSC-1 controls, with odds ratios of 0.57 (p = 0.046) and 0.41 (p = 0.0038), respectively (Fig. 1, top panel). However, after Bonferroni’s multiple comparisons adjustment, these associations did not reach significance. In contrast, MSC-1 controls had significantly higher odds of reporting that they could not afford specialist or follow-up care, or mental health counseling, compared to LSC-1 controls, with odds ratios of 2.11 (p = 0.0073), 3.34 (p = 8.74e−05), and 1.95 (p = 0.048), respectively. For secondary lung cancer patients, cases had significantly higher odds of reporting that they were somewhat or very worried about paying, compared to LSC-2 controls, with an odds ratio of 1.31 (p = 0.030) (Fig. 1, bottom panel). However, none of the odds ratios in the healthcare access analysis for secondary lung cancer patients met the stricter Bonferroni significance threshold.

### Family history patterns in primary and secondary lung cancer

**Figure 2:**
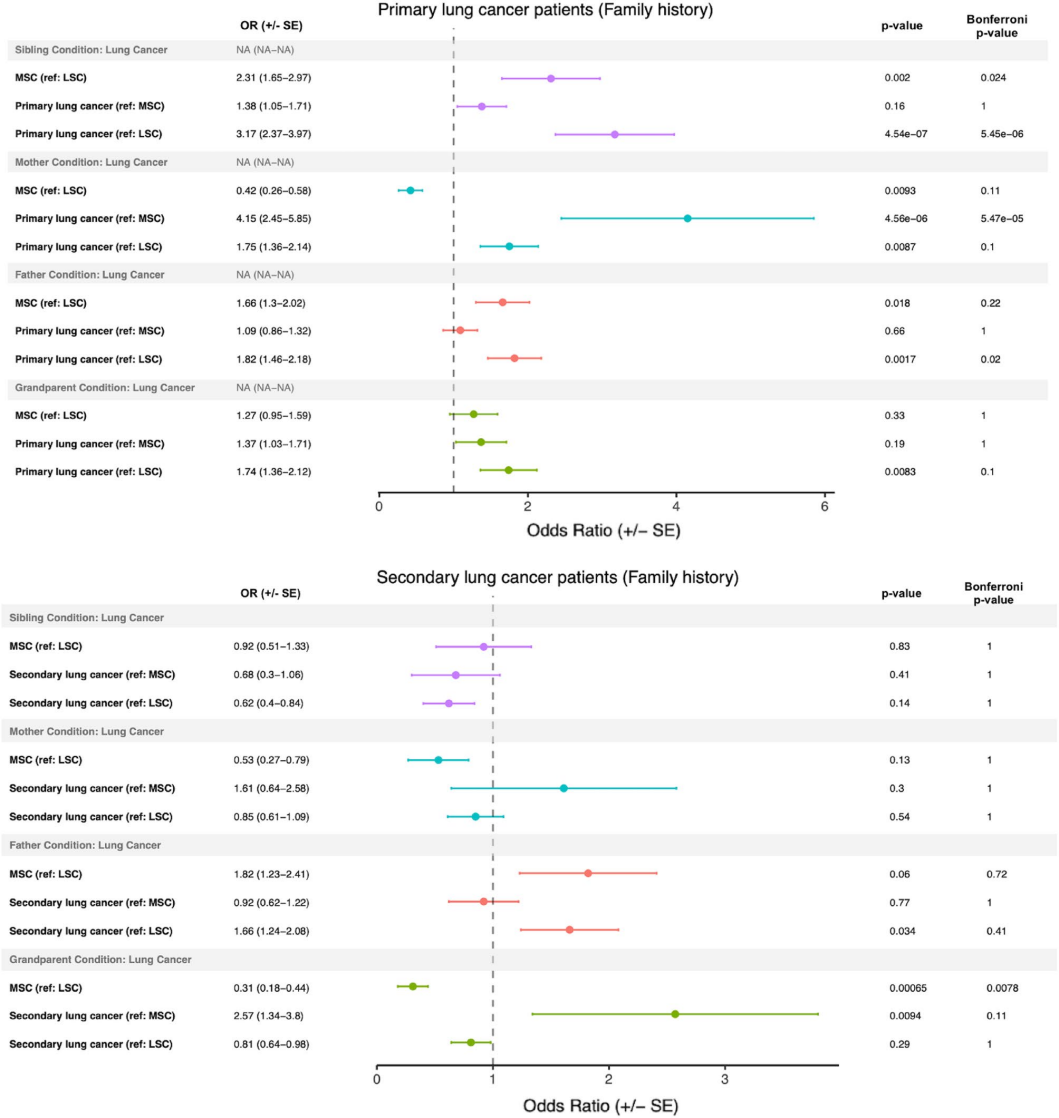
Family history in primary and secondary lung cancer patients. Odds ratios (± standard error) generated comparing family history patient-reported metrics in primary and secondary lung cancer patients to light smoking (LSC) and matched smoking (MSC) controls. The reference group (e.g., (ref: MSC)), mid-p value, and Bonferroni-corrected p-values are reported for each comparison.

In our investigation of familial history in primary and secondary lung cancer patients, we found that smoking status, rather than lung cancer diagnosis, was associated with an increased odds of having a sibling or father with primary lung cancer (Fig. 2, top panel). The odds of having a sibling with lung cancer comparing both MSC-1 controls and primary lung cancer patients with LSC-1 controls were 2.31 (p = 0.0020) and 3.17 (p = 4.54e−07), respectively, with both p-values remaining significant after Bonferroni correction. Similarly, the odds of having a father with lung cancer comparing both MSC-1 controls and primary lung cancer patients with LSC-1 controls were 1.66 (p = 0.018) and 1.82 (p = 0.0017), respectively, with the latter maintaining significance after Bonferroni correction. Although the odds of having a mother or grandparent with lung cancer were also increased when comparing our primary lung cancer patients to LSC-1 controls, with odds ratios of 1.75 (p = 0.0087) and 1.74 (p = 0.0083), respectively, neither remained significant after Bonferroni correction. For patients with secondary lung cancer, the odds of having a father with lung cancer were increased with an odds ratio of 1.66 (p = 0.034) compared to LSC-2 controls, while MSC-2 controls compared to LSC-2 controls had an odds ratio of 0.31 (p = 0.00065) of having a grandparent with lung cancer (Fig. 2, bottom panel).

### Smoking-related behavior in primary and secondary lung cancer

**Figure 3:**
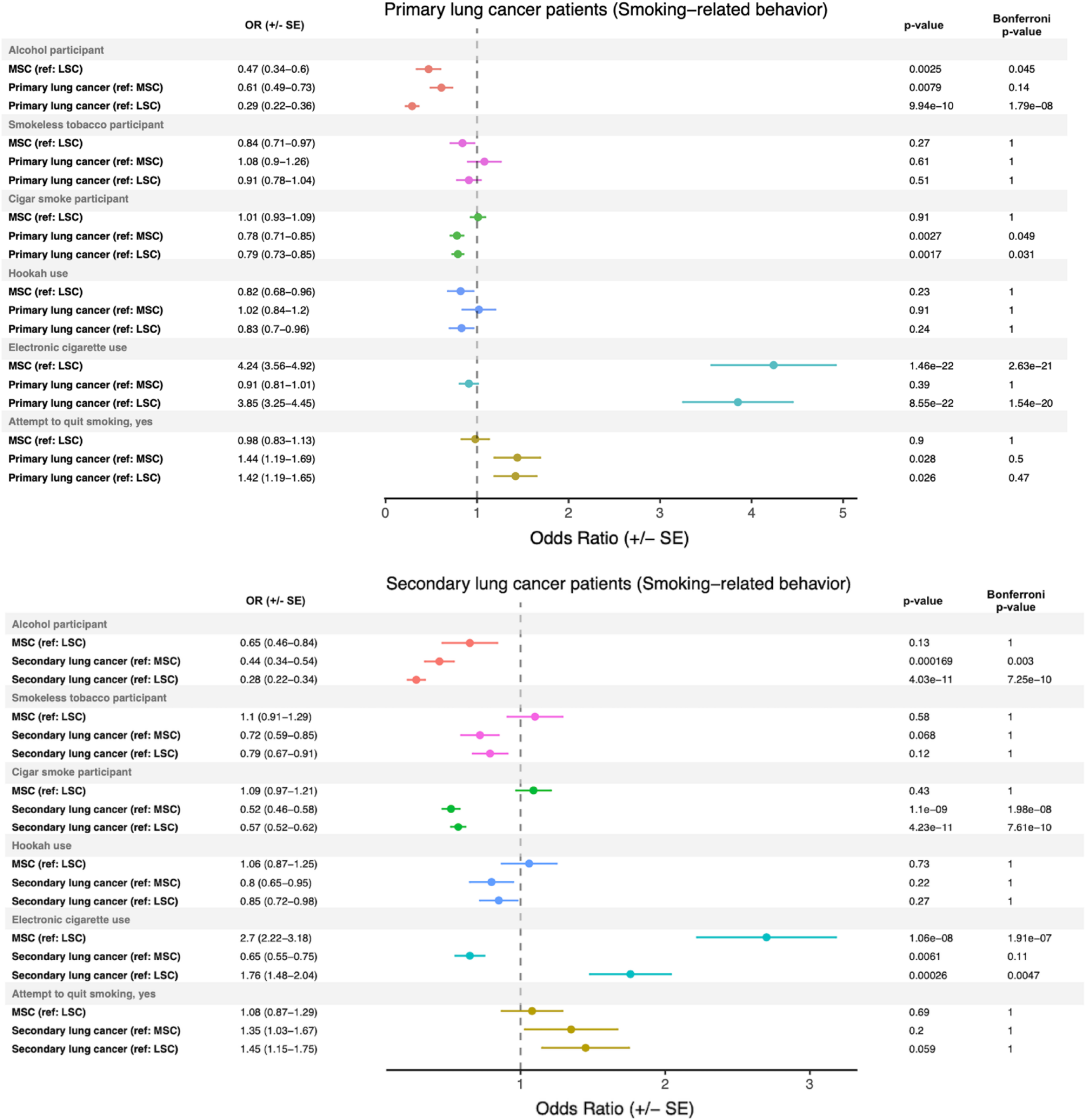
Smoking-related behaviors in primary and secondary lung cancer patients. Odds ratios (± standard error) generated comparing smoking-related behaviors from patient-reported metrics in primary and secondary lung cancer patients to light smoking (LSC) and matched smoking (MSC) controls. The reference group (e.g., (ref: MSC)), mid-p value, and Bonferroni-corrected p-values are reported for each comparison.

We investigated several smoking-related behaviors, including electronic cigarette use, smokeless tobacco use, hookah use, cigar smoking, and alcohol use, in both primary and secondary lung cancer patients. We observed that primary lung cancer patients had a significantly lower odds of using alcohol compared to all comparison groups (Fig. 3, top panel). Additionally, primary lung cancer patients had a significantly lower odds of using cigars compared to both MSC-1 and LSC-1 controls, with odds ratios of 0.78 (p = 0.0027) and 0.79 (p = 0.0017), respectively, which retained significance after Bonferroni correction. Interestingly, electronic cigarette use was found to be associated with smoking status rather than lung cancer status, with both primary lung cancer patients and MSC-1 controls having a greater odds of using electronic cigarettes compared to LSC-1 controls, with odds ratios of 3.85 (p = 8.55e−22) and 4.24 (p = 1.46e−22), respectively. These associations also retained significance after Bonferroni correction. Furthermore, primary lung cancer patients demonstrated a nominally significant increased odds of having made a serious smoking quit attempt compared to both MSC-1 and LSC-1 controls, with odds ratios of 1.44 (p = 0.028) and 1.42 (p = 0.026), respectively.

In the analysis of smoking-related behaviors in secondary lung cancer patients, both comparison groups had a Bonferroni-corrected significantly lower odds of using alcohol (Fig. 3, bottom panel). When compared to both MSC-2 and LSC-2 controls, secondary lung cancer patients demonstrated a Bonferroni-corrected significantly lower odds of using cigars, with odds ratios of 0.52 (p = 1.1e−09) and 0.57 (p = 4.23e−11), respectively. Electronic cigarette use was associated with smoking status, rather than lung cancer status. MSC-2 controls had a 2.70 greater odds (p = 1.06e−08) than LSC-2 controls, and secondary lung cancer patients had a 1.76 greater odds (p = 0.00026) than LSC-2 controls of using electronic cigarettes. These associations retained significance after Bonferroni correction.

### Primary and secondary lung cancer are associated with significant psychiatric comorbidities

**Figure 4:**
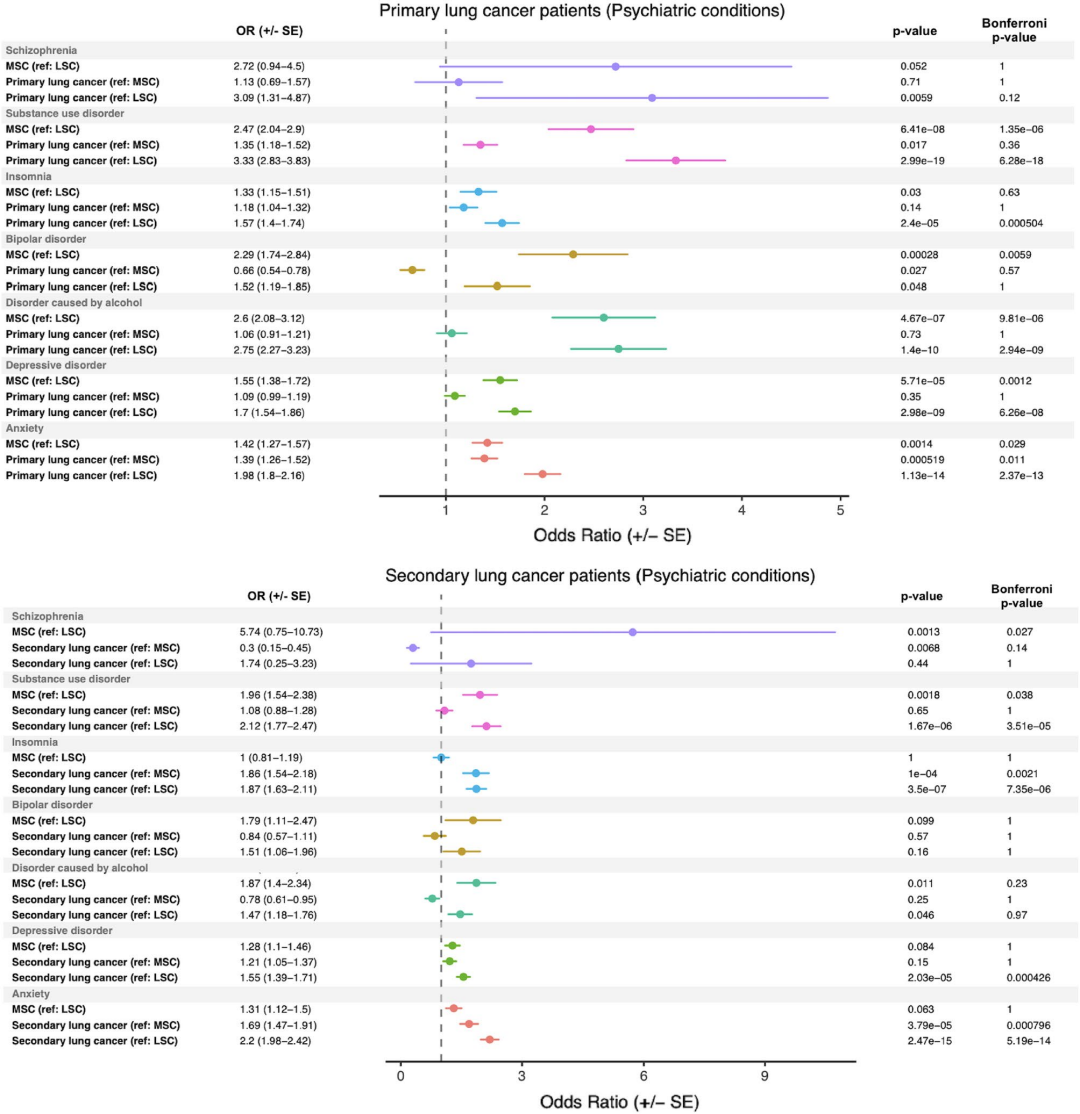
Psychiatric disease burden in primary and secondary lung cancer patients. Odds ratios (± standard error) generated comparing psychiatric disease burden from patient EHR data in primary and secondary lung cancer patients to light smoking (LSC) and matched smoking (MSC) controls. The reference group (e.g., (ref: MSC)), Fisher p-value, and Bonferroni-corrected p-values are reported for each comparison.

We investigated the odds of lung cancer patients having psychiatric conditions in their electronic health record (EHR) compared to their controls. The analyzed conditions included anxiety, bipolar disorder, depressive disorders, disorders caused by alcohol, insomnia, schizophrenia, and substance use disorder. We found that primary lung cancer patients had significantly higher odds of having substance use disorder, insomnia, bipolar disorder, disorder caused by alcohol, depressive disorder, and anxiety compared to their LSC-1 controls. Each of these odds ratios (except for bipolar disorder) remained significant after Bonferroni correction (Fig. 4, top panel). MSC-1 controls had significantly greater odds of having a substance use disorder, bipolar disorder, disorder caused by alcohol, anxiety, or a depressive disorder when compared to LSC-1 controls. Furthermore, primary lung cancer patients had significantly higher odds of having anxiety compared to MSC-1 controls (OR: 1.39; p = 0.00052). Interestingly, smoking status was associated with comorbid substance use disorder, bipolar disorder, disorder caused by alcohol, and depressive disorder, instead of primary lung cancer status. Both MSC-1 controls versus LSC-1 controls and primary lung cancer versus LSC-1 controls had a greater odds of having these psychiatric comorbidities. Additionally, secondary lung cancer patients had significantly higher odds of having substance use disorder, insomnia, and anxiety compared to their LSC-2 controls, and these odds ratios retained significance after Bonferroni multiple comparisons adjustment (Fig. 4, bottom panel). Furthermore, secondary lung cancer patients versus the MSC-2 controls had significantly higher odds of having comorbid insomnia and anxiety.

## Discussion

**Figure 5:**
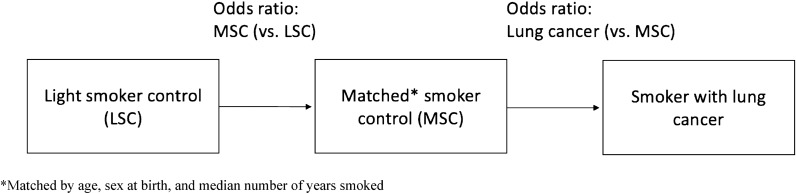
Dual control study design. Schema depicting the dual control design utilized in the present study.

In this cross-sectional, case–control study, we examined various epidemiological factors and psychiatric comorbidities in primary and secondary lung cancer. Previous case–control studies on primary lung cancer have investigated factors such as diet^29,30^, occupational exposure^31,32^, physical activity^33^, medications^34,35^, cannabis use^36^, genetic polymorphisms^37^, and various other factors. However, our study is the first to report key epidemiological information in lung cancer from the All of Us Research Program, which has a focus on recruiting historically underrepresented individuals^38^. Additionally, our dual control study design allowed us to differentiate the effect of smoking from the effect of lung cancer when examining variables of interest (Fig. 5). We investigated differences in healthcare access, family history, smoking-related behavior, and psychiatric disease burden in our cohort of primary and secondary lung cancer, as well as in the light smoking and matched smoking controls.

The issue of healthcare access and equity is of significant concern in cancer research. In a previous study, it was discovered that cancer death rates in men and women are 13% and 3% higher, respectively, in poorer counties compared to more affluent counties^39^. Furthermore, the same study found that non-Hispanic whites have higher 5-year cancer survival rates than African American, American Indian/Alaskan Native, and Asian/Pacific Islander men^39^. These findings underscore the need to identify and remove barriers to healthcare. Our study produced positive results, as none of the examined lung cancer groups reached Bonferroni-corrected levels of significance for access to care metrics such as increased worry about payment or concern about high copays or deductibles (Fig. 1). While our analysis was conducted on the entire cohort of primary and secondary lung cancer patients, future studies can stratify by race, ethnicity, and income to identify potential nuanced differences between these groups regarding access to care metrics.

Family history in primary lung cancer plays an important, yet not fully characterized, role in determining a patient’s predisposition to primary lung cancer^40^. Presently, our results demonstrate that while a first-degree relative with primary lung cancer can increase the odds of a patient having primary lung cancer (Fig. 2, top panel), family history understandably cannot explain the entire risk. Interestingly, we also saw that an increased odds of having a sibling or father with lung cancer was associated more with smoking behavior, with both primary lung cancer patients and their matched smoking controls compared to light smoking controls having a similar odds of having a sibling or father with lung cancer. This suggests a strong role for the environment in the development of lung cancer, and various literature demonstrates that smoking behavior is correlated in families^41–43^. One major limitation of our family history analysis, however, is that the data is self-reported through a survey, meaning (1) there was no stratification between primary and secondary lung cancer in relatives and (2) not all cases of familial lung cancer will be captured.

In addition to cigarette smoking, we investigated other smoking-related behaviors, such as alcohol use^44^, electronic cigarette use^14^, cigar smoking, hookah use, and smokeless tobacco use. Both primary and secondary lung cancer patients showed a lower odds of using alcohol or cigars, and secondary lung cancer patients also showed a lower odds of using smokeless tobacco compared to the MSC-2 control (Fig. 3). Interestingly, in our primary lung cancer patient analysis, electronic cigarette use was associated with smoking status, irrespective of whether or not the patient had primary lung cancer. Namely, smokers (with and without lung cancer) were more likely to use electronic cigarettes compared to their light smoking counterparts. Electronic cigarette use (i.e., vaping) has increased significantly in recent years, and smokers may view vaping as a safer alternative, which can explain the trend observed in this study^14,45^. The safety of vaping is under active investigation, and many researchers are concerned about the rapid rise in patients presenting with e-cigarette use-associated lung injury (EVALI)^45^. While vaping may be a more benign alternative to smoking, evidence strongly suggests that vaping has its own associated risks.

Finally, the presence of significant psychiatric comorbidities is well-known in cancer^46^, including lung cancer^47^. By querying electronic health records, we wanted to understand whether or not smoking and/or lung cancer increased the odds of having a comorbid psychiatric condition and by how much. The results from this analysis demonstrated that primary lung cancer patients have a significantly higher odds of having comorbid substance use disorder, insomnia, bipolar disorder, disorder caused by alcohol, depressive disorder, and anxiety when compared to their LSC-1 controls. Secondary lung cancer patients had a significantly higher odds of having substance use disorder, insomnia, and anxiety compared to their LSC-2 controls. However, smoking, rather than lung cancer, appeared to be associated with an increased odds of particular psychiatric comorbidities, such as substance use disorder, bipolar disorder, disorder caused by alcohol, and depressive disorder in primary lung cancer patients. This suggests that much of the psychiatric burden associated with lung cancer may be due to smoking status, rather than lung cancer diagnosis. Of the studied conditions, only secondary lung cancer patients versus matched smoking controls demonstrated a significantly increased odds of comorbid anxiety and insomnia conditions. Additionally, for these two conditions in secondary lung cancer, no significant differences were seen between the matched and light smoking controls, suggesting that the increase in odds was due to secondary lung cancer. Psychiatric conditions like anxiety and depression are well-documented in primary lung cancer^47^, and studies have demonstrated that the mood and anxiety symptoms in lung cancer patients may exceed those of other cancer patients as result of negative psychosocial and physical (e.g., symptom-related) factors^48^. Additionally, perceived negative stigma surrounding primary lung cancer, which is correlated with depressive and anxious symptoms, has been associated with greater psychiatric symptom severity^48^. Furthermore, lung cancer patients may have impaired pulmonary function, leading to lower quality of life (QoL) and increased psychiatric symptom severity^48,49^. Understanding the relationship between lung cancer, smoking, and psychiatric disease may help oncologists collaborate closely with mental health professionals to provide well-rounded, comprehensive care to lung cancer patients.

This study did have several limitations, primarily related to known challenges that occur with extracting data from electronic health records and patient surveys. First, in this study, some patients had EHR codes for both primary and secondary lung cancer, leading to an overlap between the primary and secondary lung cancer cohorts of ~ 300 patients. Another challenge is that secondary lung cancer had fewer smokers and fewer patients who filled out the survey indicating the number of years smoked, making it more challenging to assign a full suite of matched smoking controls. Notably, self-reported data from patient surveys may also be subject to bias or inaccuracies. Not every patient in the All of Us database has EMR data, and not all patients who have consented to provide their EMR data have all of their EMR data successfully integrated into the All of Us database, meaning there is a potential for errors or inconsistencies in the coding and categorization of EHR data. Given the size and diversity of the All of Us data network, there are several obstacles related to data integration, and the All of Us team has implemented data quality tools to regularly evaluate, quantify, and communicate about EHR data quality issues^50^. The present study’s cross-sectional design prevents us from making conclusions that establish a temporal relationship between smoking or lung cancer diagnosis and the diagnosis of a comorbid psychiatric condition. Moreover, there is a potential for confounding variables that were not included in the analysis, such as environmental exposures or other health conditions, and the limited number of variables included in the analysis may not fully capture the complex interactions between various epidemiological and clinical factors in lung cancer development and outcomes. Finally, the study’s focus on a specific population may not be representative of the general population, and this analysis should be repeated as the All of Us research program recruits more participants. Of particular note, 60% of participants in the All of Us v6 data release who completed the Basics survey identified as female.

In conclusion, our present cross-sectional, case–control study characterizes primary and secondary lung cancer in the All of Us database, providing information on demographics, healthcare access, family history, smoking-related behaviors, and psychiatric conditions. In future studies, using the vast array of data, including genetic information, present in the All of Us database, researchers can investigate deeper questions, such as probing the combined effect of genetic, environmental, clinical, and epidemiological factors on the development of lung cancer. Future studies can combine genetic models (e.g., polygenic risk scores) with models built from EHR information to improve predictions of disease development, progression, and management, and the All of Us database will be an excellent tool to help researchers answer a wide range of important questions.

## Supplementary Information

Supplementary Information.

## Data Availability

Data from this program are accessible at http://www.allofus.nih.gov, and this study was conducted on version 6 of the data utilizing the All of Us Researcher Workbench.
