# Supplementary material for: A comprehensive analysis of lung cancer highlighting epidemiological factors and psychiatric comorbidities from the All of Us Research Program
Source: Sci Rep. 2023 Jul 5;13:10852. doi: 10.1038/s41598-023-37585-0 (PMC10322929; doi:10.1038/s41598-023-37585-0)
Supplement: Supplementary file 1 — Supplementary Information. [file 41598_2023_37585_MOESM1_ESM.pdf]

Supplementary Material 1

Supplementary Table S1. Tumor subtype information for primary lung cancer patients

	Primary lung cancer (n = 1,451)
Malignant carcinoid tumor	73 (5.0)
Non-Hodgkin's lymphoma	1 (0.1)
Non-small cell carcinoma	563 (38.8)
Primary adenocarcinoma	400 (27.6)
Primary mucinous adenocarcinoma	51 (3.5)
Small cell carcinoma	220 (15.2)
Squamous cell carcinoma	65 (4.5)
Other / not categorized	78 (5.4)

Primary lung cancer source concept names from electronic health record (EHR) data

1. 'Primary malignant neoplasm of upper lobe, bronchus or lung'
2. 'Small cell carcinoma of lung'
3. 'Non-small cell lung cancer'
4. 'Overlapping malignant neoplasm of bronchus and lung'
5. 'Malignant neoplasm of overlapping sites of left bronchus and lung'
6. 'Malignant neoplasm of lower lobe, right bronchus or lung'
7. 'Malignant neoplasm of upper lobe, unspecified bronchus or lung'
8. 'Primary malignant neoplasm of left upper lobe of lung'
9. 'Malignant neoplasm of lower lobe, left bronchus or lung'
10. 'Malignant neoplasm of lower lobe, bronchus or lung'
11. 'Malignant neoplasm of upper lobe, bronchus or lung'
12. 'Malignant neoplasm of upper lobe, left bronchus or lung'
13. 'Malignant neoplasm of middle lobe, bronchus or lung'
14. 'Primary adenocarcinoma of lung'
15. 'Malignant neoplasm of overlapping sites of unspecified bronchus and lung'
16. 'Malignant neoplasm of upper lobe, right bronchus or lung'
17. 'Primary malignant neoplasm of lower lobe, bronchus or lung'
18. 'Malignant neoplasm of overlapping sites of right bronchus and lung'
19. 'Primary adenocarcinoma of lower lobe of left lung'
20. 'Primary malignant neoplasm of lung'
21. 'Hypertension complicating pregnancy, childbirth, and the puerperium'
22. 'Metastasis to lung from adenocarcinoma'
23. 'Malignant carcinoid tumor of lung'
24. 'Squamous cell carcinoma of right lung'
25. 'Primary malignant neoplasm of left lung'
26. 'Primary mucinous adenocarcinoma of lung'
27. 'Primary malignant neoplasm of bronchus of left lower lobe'
28. 'Malignant neoplasm of lower lobe, unspecified bronchus or lung'
29. 'Primary malignant neoplasm of lower lobe of left lung'

30. 'Primary adenocarcinoma of upper lobe of left lung'
31. 'Squamous cell carcinoma of bronchus in left upper lobe'
32. 'Non-Hodgkin\'s lymphoma of lung'
33. 'Primary malignant neoplasm of bronchus of left upper lobe'
34. 'Primary adenocarcinoma of upper lobe of right lung'
35. 'Squamous non-small cell lung cancer'
36. 'Primary malignant neoplasm of right lung'
37. 'Non-small cell carcinoma of lung, TNM stage 4'
38. 'Non-small cell carcinoma of lung, TNM stage 1'
39. 'Squamous cell carcinoma of left lung'
40. 'Squamous cell carcinoma of lung'
41. 'Malignant neoplasm of overlapping sites of bronchus and lung'

<u>Secondary lung cancer source concept names from electronic health record (EHR) data</u>

1. 'Secondary malignant neoplasm of left lung'
2. 'Secondary malignant neoplasm of lung'
3. 'Secondary malignant neoplasm of right lung'
4. 'Secondary malignant neoplasm of unspecified lung'
5. 'Metastasis to lung of unknown primary'
6. 'Secondary malignant neoplasm of bilateral lungs'
7. 'Metastasis to lung from adenocarcinoma'

<u>Anxiety source concept names</u>

1. 'Other stimulant use, unspecified with other stimulant-induced disorder'
2. 'Obsessive-compulsive disorder'
3. 'Other psychoactive substance use, unspecified with psychoactive substance-induced anxiety disorder'
4. 'Social phobia'
5. 'Generalized social phobia'
6. 'Unspecified acute reaction to stress'
7. 'Phobic anxiety disorder, unspecified'
8. 'Reaction to severe stress, unspecified'
9. NA
10. 'Acute stress disorder'
11. 'Phobia, unspecified'
12. 'Post-traumatic stress disorder, acute'
13. 'Other psychoactive substance abuse with unspecified psychoactive substance-induced disorder'
14. 'Post-traumatic stress disorder (PTSD)'
15. 'Nightmare disorder'
16. 'Other mixed anxiety disorders'
17. 'Acute stress reaction'
18. 'Anxiety disorder due to known physiological condition'
19. 'Anxiety about body function or health'
20. 'Burn-out'
21. 'Predominant disturbance of emotions'

22. 'Other reactions to severe stress'
23. 'Anxiety disorder'
24. 'Anxiety states'
25. 'Obsessive-compulsive disorder, unspecified'
26. 'Panic'
27. 'Generalized anxiety disorder'
28. 'Mixed disorders as reaction to stress'
29. 'Social phobia, generalized'
30. 'Panic attack'
31. 'Panic disorder'
32. 'Posttraumatic stress disorder'
33. 'Other specified phobia'
34. 'Alcohol dependence with alcohol-induced anxiety disorder'
35. 'Social phobia, unspecified'
36. 'Agoraphobia without mention of panic attacks'
37. 'Panic disorder without agoraphobia'
38. 'Obsessive-compulsive disorders'
39. 'Agoraphobia, unspecified'
40. 'Agoraphobia with panic disorder'
41. 'Other stimulant use, unspecified with unspecified stimulant-induced disorder'
42. 'Agoraphobia without panic disorder'
43. 'Other obsessive-compulsive disorder'
44. 'Other acute reactions to stress'
45. 'Claustrophobia'
46. 'Anxiety state'
47. 'Fear of flying'
48. 'Other isolated or specific phobias'
49. 'Other anxiety states'
50. 'Mixed anxiety and depressive disorder'
51. 'Overanxious disorder specific to childhood and adolescence'
52. 'Other specified anxiety disorders'
53. 'Panic disorder [episodic paroxysmal anxiety]'
54. 'Other stimulant use, unspecified, uncomplicated'
55. 'Post-traumatic stress disorder, chronic'
56. 'Anxiety state, unspecified'
57. 'Anxiety'
58. 'Anxiety disorder, unspecified'
59. 'Anxiety disorder in conditions classified elsewhere'
60. 'Post-traumatic stress disorder, unspecified'
61. 'Fear of blood'
62. 'Alcohol abuse with alcohol-induced anxiety disorder'
63. 'Dream anxiety disorder'
64. 'Anxiety attack'
65. 'Chronic post-traumatic stress disorder'
66. 'Other situational type phobia'
67. 'Other stimulant use, unspecified with stimulant-induced psychotic disorder, unspecified'
68. 'Acute situational disturbance'
69. 'Acute reaction to stress'
70. 'Separation anxiety disorder'
71. 'Panic disorder with agoraphobia'
72. 'Anxiety disorder due to a general medical condition'

73. 'Agoraphobia'
74. 'Other stimulant dependence with stimulant-induced anxiety disorder'
75. 'Alcohol use, unspecified with alcohol-induced anxiety disorder'
76. 'Flying phobia'
77. 'Other stimulant dependence with unspecified stimulant-induced disorder'
78. 'Cocaine dependence with cocaine-induced anxiety disorder'
79. 'Anxiety about behavior or performance'
80. 'Organic anxiety disorder'
81. 'Separation anxiety disorder of childhood'
82. 'Fear of injury'
83. 'Chronic anxiety'
84. 'Performance anxiety'
85. 'Other psychoactive substance dependence with psychoactive substance-induced anxiety disorder'
86. 'Other psychoactive substance abuse with psychoactive substance-induced anxiety disorder'
87. 'Nightmares associated with chronic post-traumatic stress disorder'
88. 'Other phobic anxiety disorders'
89. 'Cocaine use, unspecified with cocaine-induced anxiety disorder'
90. 'Other stimulant use, unspecified with stimulant-induced anxiety disorder'
91. 'Panic disorder with agoraphobia AND severe panic attacks'
92. 'Sedative, hypnotic or anxiolytic abuse with sedative, hypnotic or anxiolytic-induced anxiety disorder'
93. 'Social phobia, fear of public speaking'
94. 'Phobia'
95. 'Cocaine abuse with cocaine-induced anxiety disorder'
96. 'Acrophobia'
97. 'Other animal type phobia'
98. 'Fear of bridges'
99. 'Other psychoactive substance abuse with other psychoactive substance-induced disorder'
100. 'Anxiety about loss of memory'
101. 'Complex posttraumatic stress disorder'
102. 'Anxiety hyperventilation'
103. 'Sedative, hypnotic or anxiolytic abuse with intoxication, uncomplicated'
104. 'Phobic anxiety disorders'
105. 'Psychoactive substance-induced organic anxiety disorder'
106. 'Predominant psychomotor disturbance'
107. 'Sedative, hypnotic or anxiolytic abuse with intoxication, unspecified'
108. 'Separation anxiety'
109. 'Alcohol-induced anxiety disorder'
110. 'Gynephobia'
111. 'Phobic disorder'
112. 'Anxiety disorder caused by stimulant'
113. 'Stress reaction causing mixed disturbance of emotion and conduct'
114. 'Sedative, hypnotic or anxiolytic dependence with sedative, hypnotic or anxiolytic-induced anxiety disorder'
115. 'Other stimulant abuse with stimulant-induced anxiety disorder'
116. 'Simple phobia'
117. 'Other stimulant dependence with other stimulant-induced disorder'
118. 'Physical AND emotional exhaustion state'
119. 'Phobic disorders'
120. 'Anxiety disorder of adolescence'
121. 'Anxiousness (& symptom)'

122. 'Recurrent moderate major depressive disorder co-occurrent with anxiety'
123. 'Recurrent severe major depressive disorder co-occurrent with anxiety'
124. 'Panic disorder with agoraphobia AND moderate panic attacks'
125. 'No matching concept'
126. 'Arachnophobia'
127. 'Panic disorder without agoraphobia with moderate panic attacks'
128. 'Fear of nutritional disease'
129. 'Zoophobia'
130. 'Panic disorder with agoraphobia, severe agoraphobic avoidance AND severe panic attacks'
131. 'Anxiety disorder of childhood OR adolescence'
132. 'Fear of heights'
133. 'Severe anxiety (panic)'
134. 'Moderate anxiety'
135. 'School phobia'
136. 'Chronic stress disorder'
137. 'Recurrent major depressive disorder in partial remission co-occurrent with anxiety'
138. 'Parasitophobia'
139. 'Needle phobia'
140. 'Agoraphobia without history of panic disorder'
141. 'Agoraphobia with panic attacks'
142. 'Mild anxiety'
143. 'Acute fugue state due to acute stress reaction'
144. 'Posttraumatic stress disorder, delayed onset'
145. 'Acute post-trauma stress state'

Depressive disorder source concept names

1. 'Single major depressive episode'
2. 'Bipolar I disorder, most recent episode (or current) depressed, in full remission'
3. 'Major depressive disorder, single episode, severe with psychotic features'
4. 'Recurrent major depressive episodes, mild'
5. 'Recurrent major depressive episodes'
6. 'Severe depression'
7. 'Other depressive episodes'
8. 'Recurrent major depression in remission'
9. 'Premenstrual dysphoric disorder'
10. 'Bipolar I disorder, most recent episode (or current) depressed, moderate'
11. 'Bipolar I disorder, most recent episode (or current) depressed, in partial or unspecified remission'
12. 'Major depressive disorder, single episode, severe without psychotic features'
13. 'Mild depression'
14. 'Bipolar disorder, current episode depressed, moderate'
15. 'Major depressive affective disorder, single episode, severe, specified as with psychotic behavior'
16. 'Severe recurrent major depression'
17. NA
18. 'Major depressive affective disorder, single episode, moderate'
19. 'Bipolar disorder, current episode depressed, severe, with psychotic features'
20. 'Bipolar I disorder, most recent episode (or current) depressed, mild'
21. 'Bipolar disorder, current episode depressed, mild or moderate severity, unspecified'
22. 'Bipolar disorder, current episode depressed, mild'
23. 'Major depressive affective disorder, single episode, in partial or unspecified remission'

24. 'Recurrent major depression in partial remission'
25. 'Other recurrent depressive disorders'
26. 'Moderate major depression, single episode'
27. 'Major depressive affective disorder, recurrent episode, in full remission'
28. 'Single major depressive episode, in full remission'
29. 'Major depressive affective disorder, recurrent episode, severe, specified as with psychotic behavior'
30. 'Mild recurrent major depression'
31. 'Recurrent major depression in full remission'
32. 'Other specified depressive episodes'
33. 'Mixed anxiety and depressive disorder'
34. 'Major depressive affective disorder, recurrent episode, mild'
35. 'Postpartum depression'
36. 'Recurrent major depressive episodes, moderate'
37. 'Major depressive affective disorder, single episode, in full remission'
38. 'Seasonal affective disorder'
39. 'Major depressive disorder, single episode, in full remission'
40. 'Moderate recurrent major depression'
41. 'Major depressive disorder, single episode, moderate'
42. 'Reactive depression (situational)'
43. 'Schizoaffective disorder, depressive type'
44. 'Major depression in partial remission'
45. 'Bipolar I disorder, most recent episode (or current) depressed, unspecified'
46. 'Major depressive disorder, single episode, in partial remission'
47. 'Major depressive disorder, recurrent episode'
48. 'Major depressive affective disorder, single episode, unspecified'
49. 'Major depressive disorder, recurrent, severe with psychotic symptoms'
50. 'Major depressive disorder, recurrent, in partial remission'
51. 'Major depressive disorder'
52. 'Major depressive disorder, recurrent, in remission, unspecified'
53. 'Bipolar affective disorder, currently depressed, mild'
54. 'Major depressive affective disorder, single episode, severe, without mention of psychotic behavior'
55. 'Dysthymia'
56. 'Major depressive disorder, single episode, mild'
57. 'Single major depressive episode, moderate'
58. 'Dysthymic disorder'
59. 'Major depression, single episode'
60. 'Depressive disorder'
61. 'Recurrent major depression'
62. 'Major depressive affective disorder, recurrent episode, in partial or unspecified remission'
63. 'Major depressive disorder, recurrent, unspecified'
64. 'Major depressive disorder, recurrent, in full remission'
65. 'Depression, unspecified'
66. 'Single episode of major depression in full remission'
67. 'Major depressive disorder, recurrent severe without psychotic features'
68. 'Major depressive disorder, recurrent, mild'
69. 'Major depressive disorder, recurrent, moderate'
70. 'Major depressive affective disorder, single episode, mild'
71. 'Major depressive affective disorder, recurrent episode, unspecified'

72. 'Major depressive affective disorder, recurrent episode, severe, without mention of psychotic behavior'
73. 'Bipolar disorder, current episode depressed, severe, without psychotic features'
74. 'Major depressive affective disorder, recurrent episode, moderate'
75. 'Major depressive disorder, single episode, unspecified'
76. 'Severe recurrent major depression without psychotic features'
77. 'Depressive disorder, not elsewhere classified'
78. 'Chronic depressive personality disorder'
79. 'Major depression in full remission'
80. 'Major depressive disorder, recurrent'
81. 'Mild major depression'
82. 'Bipolar I disorder, most recent episode (or current) depressed'
83. 'Single major depressive episode, mild'
84. 'Atypical depressive disorder'
85. 'Major depressive disorder, single episode'
86. 'Bipolar affective disorder, current episode depression'
87. 'Severe major depression, single episode, without psychotic features'
88. 'Chronic depression'
89. 'Recurrent major depressive episodes, in full remission'
90. 'Major depression in remission'
91. 'Mild major depression, single episode'
92. 'Depressive type psychosis'
93. 'Recurrent major depressive disorder with postpartum onset'
94. 'Severe recurrent major depression with psychotic features'
95. 'Vascular dementia, with depressed mood'
96. 'Major depression with psychotic features'
97. 'Psychosis and severe depression co-occurrent and due to bipolar affective disorder'
98. 'Endogenous depression'
99. 'Severe major depression with psychotic features, mood-congruent'
100. 'Major depression single episode, in partial remission'
101. 'Recurrent depression'
102. 'Menopausal depression'
103. 'Severe major depression with psychotic features'
104. 'Acute depression'
105. 'Severe major depression'
106. 'Severe major depression, single episode'
107. 'Depressive disorder in remission'
108. 'Severe major depression, single episode, with psychotic features'
109. 'Severe postnatal depression'
110. 'Recurrent major depressive episodes, severe, with psychosis'
111. 'Multi-infarct dementia with depression'
112. 'Moderate major depression'
113. 'Bipolar affective disorder, currently depressed, moderate'
114. 'Chronic recurrent major depressive disorder'
115. 'Major depression, melancholic type'
116. 'Moderately severe major depression'
117. 'Moderately severe recurrent major depression'
118. 'Bipolar affective disorder, currently depressed, in full remission'
119. 'Moderate depression'
120. 'Single major depressive episode, severe, with psychosis'
121. 'Late onset dysthymia'

122. 'Maternity blues'
123. 'Drug-induced depressive state'
124. 'Severe major depression without psychotic features'
125. 'Depressive episode'
126. 'Major depressive disorder, recurrent, in remission'
127. 'Recurrent major depressive disorder with melancholic features'
128. 'Recurrent severe major depressive disorder co-occurrent with anxiety'
129. 'Minimal major depression'
130. 'Moderately severe depression'
131. 'Recurrent moderate major depressive disorder co-occurrent with anxiety'
132. 'Chronic major depressive disorder, single episode'
133. 'O/E - depressed'
134. 'No matching concept'
135. 'Secondary dysthymia'
136. 'Depressive disorder in mother complicating pregnancy'
137. 'Recurrent major depressive disorder in partial remission co-occurrent with anxiety'
138. 'Bipolar disorder, current episode depressed, mild or moderate severity'

<u>Disorder caused by alcohol source concept names</u>

1. 'Alcoholic polyneuropathy'
2. 'Alcohol dependence with alcohol-induced mood disorder'
3. 'Chronic alcoholism in remission'
4. 'Alcohol dependence with withdrawal delirium'
5. 'Alcohol induced acute pancreatitis with infected necrosis'
6. 'Alcohol use, unspecified with other alcohol-induced disorder'
7. 'Alcohol dependence with intoxication, unspecified'
8. 'Alcohol abuse, episodic'
9. 'Alcoholic fatty liver'
10. 'Alcohol withdrawal'
11. 'Alcohol induced acute pancreatitis without necrosis or infection'
12. 'Alcoholic liver damage, unspecified'
13. 'Alcoholic cirrhosis of liver'
14. 'Severe alcohol dependence'
15. 'Acute alcoholic hepatitis'
16. NA
17. 'Alcohol abuse'
18. 'Alcohol dependence with intoxication delirium'
19. 'Alcohol dependence with intoxication, uncomplicated'
20. 'Alcohol abuse with other alcohol-induced disorder'
21. 'Acute alcoholic intoxication in alcoholism, unspecified'
22. 'Alcohol-induced psychotic disorder with hallucinations'
23. 'Alcohol abuse, continuous'
24. 'Alcohol dependence with other alcohol-induced disorder'
25. 'Alcohol dependence with withdrawal, uncomplicated'
26. 'Alcoholic hepatitis without ascites'
27. 'Alcohol dependence'
28. 'Alcohol-induced organic mental disorder'
29. 'Alcohol-induced chronic pancreatitis'
30. 'Wernicke\'s encephalopathy'
31. 'Alcohol dependence with withdrawal with perceptual disturbance'

32. 'Alcoholic cirrhosis'
33. 'Acute alcoholic intoxication in alcoholism, continuous'
34. 'Alcohol use, unspecified with intoxication, unspecified'
35. 'Alcohol use, unspecified with intoxication, uncomplicated'
36. 'Alcohol abuse, in remission'
37. 'Alcohol dependence with alcohol-induced psychotic disorder, unspecified'
38. 'Alcohol dependence with alcohol-induced persisting dementia'
39. 'Alcohol abuse with intoxication, uncomplicated'
40. 'Alcohol dependence with alcohol-induced anxiety disorder'
41. 'Alcoholic cirrhosis of liver without ascites'
42. 'Alcohol abuse with intoxication, unspecified'
43. 'Alcohol withdrawal delirium'
44. 'Alcohol dependence with alcohol-induced psychotic disorder with hallucinations'
45. 'Other and unspecified alcohol dependence'
46. 'Alcohol induced acute pancreatitis'
47. 'Alcohol use, unspecified with alcohol-induced mood disorder'
48. 'Alcohol abuse, unspecified'
49. 'Alcohol dependence with withdrawal, unspecified'
50. 'Alcohol-induced acute pancreatitis'
51. 'Other and unspecified alcohol dependence, continuous'
52. 'Alcohol abuse with unspecified alcohol-induced disorder'
53. 'Alcohol abuse with alcohol-induced mood disorder'
54. 'Alcoholic hepatic failure without coma'
55. 'Alcoholic liver disease, unspecified'
56. 'Acute alcoholic intoxication in alcoholism, in remission'
57. 'Alcohol dependence with unspecified alcohol-induced disorder'
58. 'Alcohol use, unspecified with unspecified alcohol-induced disorder'
59. 'Alcohol dependence, in remission'
60. 'Alcoholism'
61. 'Alcohol intoxication'
62. 'Alcohol abuse, uncomplicated'
63. 'Other and unspecified alcohol dependence, in remission'
64. 'Alcohol dependence, uncomplicated'
65. 'Other and unspecified alcohol dependence, episodic'
66. 'Other and unspecified alcohol dependence, unspecified'
67. 'Alcohol use, unspecified with withdrawal, unspecified'
68. 'Unspecified alcohol-induced mental disorders'
69. 'Nondependent alcohol abuse in remission'
70. 'Alcohol induced acute pancreatitis with uninfected necrosis'
71. 'Alcohol use, unspecified with alcohol-induced anxiety disorder'
72. 'Alcohol use, unspecified with alcohol-induced persisting amnestic disorder'
73. 'Other alcohol-induced mental disorders'
74. 'Alcohol abuse with alcohol-induced anxiety disorder'
75. 'Alcohol use, unspecified with alcohol-induced psychotic disorder, unspecified'
76. 'Alcohol abuse with withdrawal, unspecified'
77. 'Alcohol abuse with alcohol-induced sleep disorder'
78. 'Alcohol-induced persisting dementia'
79. 'Alcohol abuse with intoxication delirium'
80. 'Idiosyncratic alcohol intoxication'
81. 'Alcohol use, unspecified with intoxication delirium'
82. 'Alcohol affecting fetus or newborn via placenta or breast milk'

83. 'Moderate alcohol dependence'
84. 'Alcohol abuse with alcohol-induced psychotic disorder, unspecified'
85. 'Alcohol use, unspecified with alcohol-induced psychotic disorder with hallucinations'
86. 'Alcohol use, unspecified with alcohol-induced persisting dementia'
87. 'Alcohol dependence with alcohol-induced psychotic disorder with delusions'
88. 'Accidental poisoning by alcoholic beverages'
89. 'Alcohol dependence with alcohol-induced sleep disorder'
90. 'Alcohol abuse with withdrawal, uncomplicated'
91. 'Acute alcoholic intoxication in alcoholism, episodic'
92. 'Wernicke\'s disease'
93. 'Alcohol intoxication delirium'
94. 'Persistent alcohol abuse'
95. 'Fetal alcohol syndrome (dysmorphic)'
96. 'Alcohol use, unspecified with alcohol-induced sleep disorder'
97. 'Alcohol use complicating pregnancy, second trimester'
98. 'Alcohol-induced persisting amnestic disorder'
99. 'Nondependent alcohol abuse, continuous'
100. 'Alcoholic myopathy'
101. 'Alcohol abuse with alcohol-induced psychotic disorder with hallucinations'
102. 'Acute alcoholic intoxication'
103. 'Alcohol use complicating pregnancy, unspecified trimester'
104. 'Other specified alcohol-induced mental disorders'
105. 'Alcoholic hepatitis'
106. 'Alcohol withdrawal syndrome'
107. 'Nondependent alcohol abuse, episodic'
108. 'Alcohol dependence with alcohol-induced persisting amnestic disorder'
109. 'Alcohol abuse with withdrawal with perceptual disturbance'
110. 'Alcohol use complicating childbirth'
111. 'Alcohol-induced psychotic disorder with delusions'
112. 'Alcohol induced sleep disorders'
113. 'Alcoholic fibrosis and sclerosis of liver'
114. 'Alcohol use, unspecified with withdrawal, uncomplicated'
115. 'Alcoholic liver damage'
116. 'Alcohol use complicating pregnancy, first trimester'
117. 'Alcohol abuse with alcohol-induced psychotic disorder with delusions'
118. 'Alcohol use complicating pregnancy, third trimester'
119. 'Acute alcoholic intoxication in alcoholism'
120. 'Alcohol-induced mood disorder'
121. 'Alcohol use, unspecified with alcohol-induced psychotic disorder with delusions'
122. 'Uncomplicated alcohol withdrawal'
123. 'Acute alcoholic intoxication in remission, in alcoholism'
124. 'Korsakoff\'s psychosis'
125. 'Acute alcoholic liver disease'
126. 'Nondependent alcohol abuse'
127. 'Alcohol abuse with withdrawal delirium'
128. 'Alcohol dependence with withdrawal'
129. 'Alcohol use, unspecified with intoxication'
130. 'Alcohol-induced anxiety disorder'
131. 'Continuous chronic alcoholism'
132. 'Alcohol use, unspecified'
133. 'Alcohol-induced psychosis'

134. 'Alcohol-induced sleep disorder'
135. 'Alcohol-induced pancreatitis'
136. 'Alcohol use complicating the puerperium'
137. 'Newborn affected by maternal use of alcohol'
138. 'No matching concept'
139. 'Alcoholic steatohepatitis'
140. 'Episodic acute alcoholic intoxication in alcoholism'
141. 'Alcohol abuse, with withdrawal'
142. 'Alcohol hallucinosis'
143. 'Alcohol use, unspecified with withdrawal delirium'
144. 'Continuous acute alcoholic intoxication in alcoholism'
145. 'Alcohol induced disorder co-occurrent and due to alcohol dependence'
146. 'Alcohol dependence syndrome'
147. 'Alcohol amnestic disorder'
148. 'Disorder caused by alcohol'
149. 'Fetal alcohol syndrome'
150. 'Insomnia caused by alcohol'
151. 'Alcohol abuse with intoxication'
152. 'Alcoholic liver disease'
153. 'Alcohol dependence with intoxication'
154. 'Alcoholic hepatic failure'
155. 'Episodic chronic alcoholism'
156. 'Dementia associated with alcoholism'

Bipolar disorder source concept names

1. 'Bipolar I disorder, most recent episode (or current) mixed'
2. 'Bipolar affective disorder, current episode mixed'
3. 'Bipolar I disorder, most recent episode (or current) mixed, in full remission'
4. 'Bipolar disorder, in full remission, most recent episode manic'
5. 'Bipolar disorder, current episode mixed, moderate'
6. 'Bipolar I disorder, most recent episode (or current) mixed, severe, specified as with psychotic behavior'
7. 'Bipolar disorder, current episode manic without psychotic features, severe'
8. 'Bipolar disorder, current episode depressed, moderate'
9. 'Bipolar I disorder, most recent episode (or current) manic, in partial or unspecified remission'
10. 'Bipolar disorder, current episode manic without psychotic features, moderate'
11. 'Bipolar disorder'
12. 'Bipolar disorder, in partial remission, most recent episode manic'
13. 'Bipolar I disorder, most recent episode (or current) mixed, severe, without mention of psychotic behavior'
14. 'Bipolar disorder, current episode mixed, unspecified'
15. 'Bipolar disorder, current episode manic without psychotic features, mild'
16. 'Schizoaffective disorder, bipolar type'
17. NA
18. 'Bipolar disorder, in full remission, most recent episode mixed'
19. 'Bipolar I disorder'
20. 'Bipolar I disorder, most recent episode (or current) mixed, mild'
21. 'Bipolar disorder, current episode hypomanic'
22. 'Bipolar I disorder, most recent episode (or current) manic, in full remission'
23. 'Bipolar I disorder, most recent episode (or current) manic, mild'

24. 'Bipolar I disorder, single manic episode, moderate'
25. 'Bipolar I disorder, most recent episode (or current) depressed, mild'
26. 'Bipolar disorder, current episode depressed, severe, with psychotic features'
27. 'Bipolar I disorder, most recent episode (or current) depressed, severe, specified as with psychotic behavior'
28. 'Depressed bipolar I disorder in full remission'
29. 'Bipolar I disorder, most recent episode (or current) manic, severe, specified as with psychotic behavior'
30. 'Bipolar I disorder, most recent episode (or current) manic, unspecified'
31. 'Bipolar affective disorder, currently manic, in full remission'
32. 'Bipolar disorder, current episode mixed, mild'
33. 'Bipolar disorder in remission'
34. 'Bipolar disorder, current episode manic without psychotic features, unspecified'
35. 'Bipolar disorder, current episode depressed, severe, without psychotic features'
36. 'Bipolar disorder, current episode mixed, severe, with psychotic features'
37. 'Bipolar disorder, in full remission, most recent episode hypomanic'
38. 'Bipolar I disorder, most recent episode (or current) depressed, in full remission'
39. 'Bipolar disorder, in partial remission, most recent episode depressed'
40. 'Other bipolar disorder'
41. 'Bipolar disorder, in partial remission, most recent episode mixed'
42. 'Bipolar I disorder, most recent episode (or current) mixed, moderate'
43. 'Bipolar disorder, in full remission, most recent episode depressed'
44. 'Bipolar I disorder, most recent episode (or current) manic, moderate'
45. 'Bipolar disorder, current episode manic severe with psychotic features'
46. 'Bipolar I disorder, most recent episode (or current) depressed, moderate'
47. 'Bipolar I disorder, most recent episode (or current) mixed, unspecified'
48. 'Bipolar disorder, in partial remission, most recent episode hypomanic'
49. 'Bipolar II disorder'
50. 'Mild manic bipolar I disorder'
51. 'Bipolar disorder, currently in remission, most recent episode unspecified'
52. 'Bipolar disorder, current episode depressed, mild'
53. 'Bipolar disorder, current episode depressed, mild or moderate severity, unspecified'
54. 'Bipolar I disorder, most recent episode (or current) depressed, unspecified'
55. 'Other bipolar disorders'
56. 'Bipolar I disorder, most recent episode (or current) depressed, severe, without mention of psychotic behavior'
57. 'Bipolar disorder in partial remission'
58. 'Bipolar II disorder, most recent episode major depressive'
59. 'Bipolar I disorder, most recent episode (or current) depressed, in partial or unspecified remission'
60. 'Bipolar I disorder, single manic episode, unspecified'
61. 'Bipolar I disorder, most recent episode (or current) unspecified'
62. 'Bipolar disorder, current episode mixed, severe, without psychotic features'
63. 'Bipolar disorder, unspecified'
64. 'Bipolar affective disorder, current episode depression'
65. 'Bipolar I disorder, single manic episode, in partial or unspecified remission'
66. 'Bipolar I disorder, most recent episode (or current) mixed, in partial or unspecified remission'
67. 'Bipolar I disorder, most recent episode (or current) manic, severe, without mention of psychotic behavior'
68. 'Depressed bipolar I disorder in partial remission'
69. 'Bipolar I disorder, single manic episode, severe, specified as with psychotic behavior'
70. 'Bipolar affective disorder, currently depressed, mild'

71. 'Bipolar I disorder, most recent episode (or current) depressed'
72. 'Bipolar I disorder, single manic episode, mild'
73. 'Bipolar type II disorder currently in full remission'
74. 'Depressed bipolar I disorder'
75. 'Mixed bipolar affective disorder'
76. 'Moderate manic bipolar I disorder'
77. 'Bipolar affective disorder, currently manic, moderate'
78. 'Bipolar I disorder, most recent episode (or current) manic'
79. 'Severe depressed bipolar I disorder without psychotic features'
80. 'Mild bipolar II disorder, most recent episode major depressive'
81. 'Mixed bipolar affective disorder, mild'
82. 'Bipolar I disorder, most recent episode hypomanic'
83. 'Severe manic bipolar I disorder without psychotic features'
84. 'Mixed bipolar I disorder in remission'
85. 'Manic bipolar I disorder in partial remission'
86. 'Bipolar I disorder, single manic episode, severe, without mention of psychotic behavior'
87. 'Mixed bipolar affective disorder, moderate'
88. 'Bipolar affective disorder, current episode manic'
89. 'Bipolar affective disorder, currently depressed, moderate'
90. 'Mixed bipolar I disorder in partial remission'
91. 'Other and unspecified bipolar disorders'
92. 'Mild mixed bipolar I disorder'
93. 'Bipolar affective disorder, currently manic, severe, with psychosis'
94. 'Bipolar II disorder, most recent episode hypomanic'
95. 'Moderate depressed bipolar I disorder'
96. 'Mixed bipolar I disorder in full remission'
97. 'Severe bipolar disorder'
98. 'Manic bipolar I disorder in full remission'
99. 'Manic bipolar I disorder'
100. 'Bipolar affective disorder, currently depressed, in full remission'
101. 'Depressed bipolar I disorder in remission'
102. 'Bipolar I disorder, single manic episode, in full remission'
103. 'Severe manic bipolar I disorder with psychotic features'
104. 'Bipolar II disorder, most recent episode major depressive with postpartum onset'
105. 'Moderate mixed bipolar I disorder'
106. 'Psychosis and severe depression co-occurrent and due to bipolar affective disorder'
107. 'Mild depressed bipolar I disorder'
108. 'Mixed bipolar affective disorder, severe, with psychosis'
109. 'Bipolar I disorder, most recent episode depression'
110. 'Moderate bipolar II disorder, most recent episode major depressive'
111. 'Bipolar I disorder, single manic episode'
112. 'Bipolar disorder in full remission'
113. 'No matching concept'
114. 'Mild bipolar disorder'
115. 'Bipolar I disorder, most recent episode manic'
116. 'Severe mixed bipolar I disorder with psychotic features'
117. 'Severe depressed bipolar I disorder'
118. 'Bipolar disorder, current episode depressed, mild or moderate severity'
119. 'Bipolar affective disorder, most recent episode mixed'
120. 'Severe bipolar II disorder'
121. 'Bipolar II disorder, most recent episode major depressive with melancholic features'

122. 'Bipolar disorder, current episode mixed'
123. 'Bipolar type I disorder currently in full remission'
124. 'Bipolar disorder, most recent episode manic'
125. 'Mixed bipolar I disorder'
126. 'Rapid cycling bipolar II disorder'
127. 'Severe manic bipolar I disorder'
128. 'Bipolar affective disorder, currently manic, mild'
129. 'Bipolar disorder, most recent episode depression'
130. 'Severe depressed bipolar I disorder with psychotic features'

<u>Insomnia source concept names</u>

1. 'Adjustment insomnia'
2. 'Psychophysiologic insomnia'
3. 'Initial insomnia'
4. 'Insomnia'
5. 'Insomnia due to other mental disorder'
6. 'Insomnia due to mental disorder'
7. 'Other insomnia not due to a substance or known physiological condition'
8. 'Organic insomnia, unspecified'
9. 'Primary insomnia'
10. 'Chronic insomnia'
11. 'Persistent insomnia'
12. 'Insufficient sleep syndrome'
13. 'Insomnia due to medical condition'
14. 'Insomnia with sleep apnea, unspecified'
15. 'Other insomnia'
16. 'Insomnia co-occurrent and due to medical condition'
17. 'Insomnia, unspecified'
18. 'Not getting enough sleep'
19. NA
20. 'Insomnia due to medical condition classified elsewhere'
21. 'Insomnia with sleep apnea'
22. 'Insomnia disorder related to known organic factor'
23. 'Insomnia disorder related to another mental disorder'
24. 'Nonorganic insomnia'
25. 'Drug-induced insomnia'
26. 'Other organic insomnia'
27. 'Transient insomnia'
28. 'Middle insomnia'
29. 'Late insomnia'
30. 'Behavioral insomnia of childhood, sleep-onset association type'
31. 'Insomnia due to anxiety and fear'
32. 'Mixed insomnia'
33. 'Terminal insomnia'
34. 'Organic disorders of initiating and maintaining sleep [Organic insomnia]'
35. 'Behavioral insomnia of childhood'
36. 'Acute insomnia'
37. 'No matching concept'
38. 'Behavioral insomnia of childhood, unspecified type'
39. 'Behavioral insomnia of childhood, combined type'

40. 'Insomnia caused by alcohol'

<u>Substance use disorder source concept names</u>

1. 'Alcohol dependence'
2. 'Alcohol-induced organic mental disorder'
3. 'Nicotine-induced organic mental disorder'
4. 'Cocaine dependence with cocaine-induced psychotic disorder with delusions'
5. 'Other psychoactive substance abuse with unspecified psychoactive substance-induced disorder'
6. 'Alcohol abuse with other alcohol-induced disorder'
7. 'Alcohol dependence with intoxication, uncomplicated'
8. NA
9. 'Alcohol abuse with alcohol-induced anxiety disorder'
10. 'Opioid use, unspecified with other opioid-induced disorder'
11. 'Other stimulant use, unspecified with stimulant-induced psychotic disorder, unspecified'
12. 'Alcohol use, unspecified with alcohol-induced psychotic disorder, unspecified'
13. 'Sedative, hypnotic, or anxiolytic use, unspecified, uncomplicated'
14. 'Nicotine dependence, cigarettes, with unspecified nicotine-induced disorders'
15. 'Severe alcohol dependence'
16. 'Cocaine dependence with withdrawal'
17. 'Alcohol abuse, episodic'
18. 'Alcohol use complicating pregnancy, first trimester'
19. 'Other psychoactive substance dependence with withdrawal delirium'
20. 'Alcohol dependence with alcohol-induced anxiety disorder'
21. 'Alcohol abuse with alcohol-induced psychotic disorder with delusions'
22. 'Other psychoactive substance use, unspecified with psychoactive substance-induced mood disorder'
23. 'Alcohol abuse with intoxication, uncomplicated'
24. 'Opioid withdrawal'
25. 'Cocaine dependence with intoxication, unspecified'
26. 'Alcohol dependence with alcohol-induced persisting amnestic disorder'
27. 'Alcohol abuse with intoxication delirium'
28. 'Cannabis use, unspecified with unspecified cannabis-induced disorder'
29. 'Alcohol dependence with alcohol-induced persisting dementia'
30. 'Other psychoactive substance use, unspecified, uncomplicated'
31. 'Cocaine use, unspecified with intoxication, unspecified'
32. 'Alcohol withdrawal'
33. 'Other psychoactive substance dependence with other psychoactive substance-induced disorder'
34. 'Nicotine dependence unspecified, with withdrawal'
35. 'Nicotine dependence, other tobacco product, with other nicotine-induced disorders'
36. 'Other psychoactive substance use, unspecified with other psychoactive substance-induced disorder'
37. 'Alcohol use, unspecified with alcohol-induced mood disorder'
38. 'Alcohol dependence with unspecified alcohol-induced disorder'
39. 'Alcohol abuse'
40. 'Chronic alcoholism in remission'
41. 'Alcohol abuse, in remission'
42. 'Sedative, hypnotic or anxiolytic use, unspecified with unspecified sedative, hypnotic or anxiolytic-induced disorder'
43. 'Cocaine abuse with intoxication, unspecified'
44. 'Other stimulant use, unspecified with unspecified stimulant-induced disorder'

45. 'Opioid abuse with other opioid-induced disorder'
46. 'Nicotine dependence, cigarettes, with other nicotine-induced disorders'
47. 'Cocaine use, unspecified with unspecified cocaine-induced disorder'
48. 'Alcohol abuse, continuous'
49. 'Alcohol dependence with other alcohol-induced disorder'
50. 'Alcohol dependence with intoxication, unspecified'
51. 'Other psychoactive substance dependence with withdrawal, unspecified'
52. 'Alcohol dependence with alcohol-induced mood disorder'
53. 'Opioid use, unspecified with unspecified opioid-induced disorder'
54. 'Other psychoactive substance abuse with psychoactive substance-induced mood disorder'
55. 'Cocaine dependence with cocaine-induced psychotic disorder with hallucinations'
56. 'Psychoactive substance use disorder'
57. 'Alcohol abuse, unspecified'
58. 'Other psychoactive substance abuse with psychoactive substance-induced psychotic disorder, unspecified'
59. 'Nicotine dependence, cigarettes, with withdrawal'
60. 'Cocaine dependence with intoxication, uncomplicated'
61. 'Sedative, hypnotic or anxiolytic abuse, uncomplicated'
62. 'Alcohol abuse with alcohol-induced mood disorder'
63. 'Other psychoactive substance dependence, uncomplicated'
64. 'Opioid abuse with unspecified opioid-induced disorder'
65. 'Alcohol dependence with withdrawal with perceptual disturbance'
66. 'Cocaine abuse with cocaine-induced mood disorder'
67. 'Cocaine abuse with unspecified cocaine-induced disorder'
68. 'Alcohol abuse with intoxication, unspecified'
69. 'Alcohol use, unspecified with intoxication delirium'
70. 'Other psychoactive substance use, unspecified with psychoactive substance-induced anxiety disorder'
71. 'Alcohol dependence with withdrawal, uncomplicated'
72. 'Opioid dependence with other opioid-induced disorder'
73. 'Nicotine dependence, unspecified, with unspecified nicotine-induced disorders'
74. 'Other alcohol-induced mental disorders'
75. 'Opioid dependence with unspecified opioid-induced disorder'
76. 'Alcohol dependence with intoxication delirium'
77. 'Opioid dependence with opioid-induced mood disorder'
78. 'Other and unspecified alcohol dependence, continuous'
79. 'Alcohol abuse with unspecified alcohol-induced disorder'
80. 'Other and unspecified alcohol dependence, in remission'
81. 'Other stimulant use, unspecified, uncomplicated'
82. 'Sedative, hypnotic or anxiolytic dependence with sedative, hypnotic or anxiolytic-induced mood disorder'
83. 'Opioid use, unspecified, uncomplicated'
84. 'Alcohol dependence with withdrawal delirium'
85. 'Cocaine dependence with cocaine-induced mood disorder'
86. 'Alcohol abuse with withdrawal, unspecified'
87. 'Alcohol dependence with withdrawal, unspecified'
88. 'Other psychoactive substance dependence with psychoactive substance-induced mood disorder'
89. 'Cannabis use, unspecified with other cannabis-induced disorder'
90. 'Hallucinogen use, unspecified with hallucinogen-induced psychotic disorder, unspecified'
91. 'Cocaine use, unspecified, uncomplicated'
92. 'Cannabis use, unspecified, uncomplicated'

93. 'Alcohol dependence, in remission'
94. 'Other and unspecified alcohol dependence, unspecified'
95. 'Alcohol dependence, uncomplicated'
96. 'Cocaine use, unspecified with cocaine-induced psychotic disorder with hallucinations'
97. 'Other and unspecified alcohol dependence, episodic'
98. 'Alcohol abuse, uncomplicated'
99. 'Opioid dependence with withdrawal'
100. 'Moderate alcohol dependence'
101. 'Alcohol-induced psychotic disorder with hallucinations'
102. 'Cannabis dependence with psychotic disorder, unspecified'
103. 'Other psychoactive substance use, unspecified with withdrawal, uncomplicated'
104. 'Cocaine use, unspecified with cocaine-induced mood disorder'
105. 'Alcohol dependence with alcohol-induced sleep disorder'
106. 'Alcohol withdrawal delirium'
107. 'Other psychoactive substance dependence with unspecified psychoactive substance-induced disorder'
108. 'Other psychoactive substance use, unspecified with unspecified psychoactive substance-induced disorder'
109. 'Alcohol-induced persisting dementia'
110. 'Cocaine dependence with cocaine-induced psychotic disorder, unspecified'
111. 'Other psychoactive substance use, unspecified with psychoactive substance-induced psychotic disorder, unspecified'
112. 'Cannabis abuse with intoxication delirium'
113. 'Alcohol intoxication delirium'
114. 'Alcohol use, unspecified with alcohol-induced persisting amnestic disorder'
115. 'Cocaine dependence with unspecified cocaine-induced disorder'
116. 'Other stimulant use, unspecified with other stimulant-induced disorder'
117. 'Opioid use, unspecified with withdrawal'
118. 'Alcohol use complicating pregnancy, second trimester'
119. 'Other psychoactive substance use, unspecified with withdrawal, unspecified'
120. 'Other and unspecified alcohol dependence'
121. 'Alcoholism'
122. 'Cocaine use, unspecified with intoxication, uncomplicated'
123. 'Cannabis abuse with cannabis-induced anxiety disorder'
124. 'Other psychoactive substance dependence with psychoactive substance-induced anxiety disorder'
125. 'Alcohol dependence with alcohol-induced psychotic disorder, unspecified'
126. 'Hallucinogen use, unspecified, uncomplicated'
127. 'Alcohol use, unspecified with alcohol-induced psychotic disorder with hallucinations'
128. 'Other psychoactive substance abuse with psychoactive substance-induced psychotic disorder with delusions'
129. 'Psychoactive substance-induced organic hallucinosis'
130. 'Unspecified alcohol-induced mental disorders'
131. 'Other psychoactive substance dependence with withdrawal, uncomplicated'
132. 'Cocaine abuse with cocaine-induced psychotic disorder with hallucinations'
133. 'Sedative, hypnotic or anxiolytic abuse with sedative, hypnotic or anxiolytic-induced psychotic disorder with hallucinations'
134. 'Other psychoactive substance dependence with intoxication, unspecified'
135. 'Alcohol withdrawal syndrome'
136. 'Cannabis abuse with other cannabis-induced disorder'

137. 'Sedative, hypnotic or anxiolytic dependence with sedative, hypnotic or anxiolytic-induced anxiety disorder'
138. 'Other specified alcohol-induced mental disorders'
139. 'Sedative, hypnotic or anxiolytic dependence with other sedative, hypnotic or anxiolytic-induced disorder'
140. 'Nicotine dependence, chewing tobacco, with unspecified nicotine-induced disorders'
141. 'Alcohol use, unspecified with alcohol-induced sleep disorder'
142. 'Nicotine dependence, other tobacco product, with unspecified nicotine-induced disorders'
143. 'Alcohol dependence with alcohol-induced psychotic disorder with hallucinations'
144. 'Nondependent alcohol abuse in remission'
145. 'Alcohol use, unspecified with withdrawal, uncomplicated'
146. 'Cocaine abuse with cocaine-induced anxiety disorder'
147. 'Alcohol abuse with withdrawal, uncomplicated'
148. 'Cocaine dependence with other cocaine-induced disorder'
149. 'Alcohol use, unspecified with alcohol-induced anxiety disorder'
150. 'Cannabis abuse with psychotic disorder, unspecified'
151. 'Cocaine abuse with cocaine-induced psychotic disorder, unspecified'
152. 'Cocaine abuse with cocaine-induced psychotic disorder with delusions'
153. 'Cocaine abuse with intoxication, uncomplicated'
154. 'Opioid abuse with withdrawal'
155. 'Cannabis dependence with cannabis-induced anxiety disorder'
156. 'Opioid use, unspecified with opioid-induced mood disorder'
157. 'Hallucinogen dependence with hallucinogen persisting perception disorder (flashbacks)'
158. 'Cannabis use, unspecified with anxiety disorder'
159. 'Other psychoactive substance use, unspecified with withdrawal delirium'
160. 'Other psychoactive substance abuse with psychoactive substance-induced anxiety disorder'
161. 'Other stimulant use, unspecified with stimulant-induced mood disorder'
162. 'Alcohol use, unspecified with alcohol-induced persisting dementia'
163. 'Alcohol use, unspecified with withdrawal, unspecified'
164. 'Alcohol abuse with alcohol-induced psychotic disorder with hallucinations'
165. 'Persistent alcohol abuse'
166. 'Cannabis abuse with unspecified cannabis-induced disorder'
167. 'Nondependent alcohol abuse, continuous'
168. 'Alcohol dependence with alcohol-induced psychotic disorder with delusions'
169. 'Alcohol abuse with alcohol-induced sleep disorder'
170. 'Opioid dependence with opioid-induced psychotic disorder with hallucinations'
171. 'Cocaine abuse with other cocaine-induced disorder'
172. 'Nondependent alcohol abuse, episodic'
173. 'Other stimulant dependence with stimulant-induced anxiety disorder'
174. 'Hallucinogen abuse with intoxication with perceptual disturbance'
175. 'Alcohol abuse with withdrawal delirium'
176. 'Psychoactive substance-induced organic mood disorder'
177. 'Cocaine use, unspecified with cocaine-induced psychotic disorder, unspecified'
178. 'Alcohol abuse with alcohol-induced psychotic disorder, unspecified'
179. 'Hallucinogen use, unspecified with unspecified hallucinogen-induced disorder'
180. 'Cocaine abuse with intoxication with perceptual disturbance'
181. 'Sedative, hypnotic AND/OR anxiolytic-related disorder'
182. 'Sedative, hypnotic or anxiolytic use, unspecified with intoxication, unspecified'
183. 'Hallucinogen use, unspecified with intoxication, uncomplicated'

184. 'Other psychoactive substance use, unspecified with intoxication with delirium'
185. 'Cocaine dependence with cocaine-induced anxiety disorder'
186. 'Cannabis dependence with unspecified cannabis-induced disorder'
187. 'Psychoactive substance-induced organic mental disorder'
188. 'Cocaine abuse with intoxication with delirium'
189. 'Alcohol-induced anxiety disorder'
190. 'Other psychoactive substance abuse with other psychoactive substance-induced disorder'
191. 'Hallucinogen use, unspecified with intoxication, unspecified'
192. 'Opioid abuse with opioid-induced mood disorder'
193. 'Alcohol-induced persisting amnestic disorder'
194. 'Nicotine dependence, chewing tobacco, with other nicotine-induced disorders'
195. 'Sedative, hypnotic or anxiolytic abuse with sedative, hypnotic or anxiolytic-induced anxiety disorder'
196. 'Other stimulant use, unspecified with stimulant-induced anxiety disorder'
197. 'Alcohol use complicating pregnancy, unspecified trimester'
198. 'Sedative, hypnotic or anxiolytic use, unspecified with other sedative, hypnotic or anxiolytic-induced disorder'
199. 'Cannabis dependence with other cannabis-induced disorder'
200. 'Opioid use, unspecified with intoxication delirium'
201. 'Cocaine use, unspecified with intoxication with perceptual disturbance'
202. 'Psychoactive substance dependence'
203. 'Hallucinogen abuse with intoxication, uncomplicated'
204. 'Sedative, hypnotic or anxiolytic dependence with intoxication delirium'
205. 'Sedative, hypnotic or anxiolytic use, unspecified with sedative, hypnotic or anxiolytic-induced sleep disorder'
206. 'Other psychoactive substance dependence with intoxication, uncomplicated'
207. 'Sedative, hypnotic or anxiolytic abuse with sedative, hypnotic or anxiolytic-induced mood disorder'
208. 'Cannabis use, unspecified with intoxication delirium'
209. 'Alcohol abuse with withdrawal with perceptual disturbance'
210. 'Other psychoactive substance abuse with intoxication delirium'
211. 'Sedative, hypnotic or anxiolytic use, unspecified with sedative, hypnotic or anxiolytic-induced mood disorder'
212. 'Opioid intoxication delirium'
213. 'Cocaine dependence with intoxication delirium'
214. 'Hallucinogen dependence with intoxication, unspecified'
215. 'Idiosyncratic alcohol intoxication'
216. 'Cocaine abuse, unspecified with withdrawal'
217. 'Opioid dependence with opioid-induced psychotic disorder with delusions'
218. 'Opioid-induced organic mental disorder'
219. 'Other psychoactive substance use, unspecified with psychoactive substance-induced persisting amnestic disorder'
220. 'Cocaine use, unspecified with intoxication delirium'
221. 'Cocaine use, unspecified with other cocaine-induced disorder'
222. 'Other psychoactive substance dependence with psychoactive substance-induced psychotic disorder with hallucinations'
223. 'Other stimulant dependence with unspecified stimulant-induced disorder'
224. 'Other psychoactive substance dependence with psychoactive substance-induced sexual dysfunction'
225. 'Cannabis use, unspecified with psychotic disorder with hallucinations'
226. 'Other stimulant dependence with other stimulant-induced disorder'

227. 'Nicotine dependence, unspecified, with other nicotine-induced disorders'
228. 'Opioid use, unspecified with opioid-induced psychotic disorder, unspecified'
229. 'Opioid dependence with intoxication delirium'
230. 'Other stimulant abuse with stimulant-induced anxiety disorder'
231. 'Opioid use, unspecified with opioid-induced sexual dysfunction'
232. 'Cannabis-induced organic mental disorder'
233. 'Nondependent alcohol abuse'
234. 'Uncomplicated alcohol withdrawal'
235. 'Continuous chronic alcoholism'
236. 'Alcohol use, unspecified with alcohol-induced psychotic disorder with delusions'
237. 'Other psychoactive substance dependence with psychoactive substance-induced psychotic disorder with delusions'
238. 'Other psychoactive substance dependence with psychoactive substance-induced psychotic disorder, unspecified'
239. 'Hallucinogen use, unspecified with hallucinogen-induced mood disorder'
240. 'Opioid dependence with opioid-induced psychotic disorder, unspecified'
241. 'Cannabis abuse with psychotic disorder with hallucinations'
242. 'Sedative, hypnotic or anxiolytic abuse with intoxication, unspecified'
243. 'Opioid abuse with opioid-induced psychotic disorder, unspecified'
244. 'Opioid abuse with intoxication delirium'
245. 'Psychoactive substance-induced withdrawal syndrome'
246. 'Other psychoactive substance abuse with psychoactive substance-induced psychotic disorder with hallucinations'
247. 'Hallucinogen use, unspecified with intoxication with delirium'
248. 'Amphetamine-induced psychotic disorder with hallucinations'
249. 'Other psychoactive substance abuse with withdrawal, unspecified'
250. 'Cannabis use, unspecified with psychotic disorder, unspecified'
251. 'Cocaine dependence with intoxication with perceptual disturbance'
252. 'Cocaine use, unspecified with cocaine-induced anxiety disorder'
253. 'Sedative, hypnotic or anxiolytic use, unspecified with sedative, hypnotic or anxiolytic-induced persisting amnestic disorder'
254. 'Sedative, hypnotic or anxiolytic use, unspecified with intoxication delirium'
255. 'Hallucinogen abuse with intoxication, unspecified'
256. 'Sedative, hypnotic or anxiolytic abuse with intoxication, uncomplicated'
257. 'Cocaine use, unspecified with withdrawal'
258. 'Psychoactive substance-induced organic anxiety disorder'
259. 'Hallucinogen use, unspecified with hallucinogen-induced psychotic disorder with hallucinations'
260. 'Alcohol-induced psychosis'
261. 'Sedative, hypnotic or anxiolytic-related abuse'
262. 'Alcohol-induced sleep disorder'
263. 'Alcohol induced sleep disorders'
264. 'Sedative, hypnotic or anxiolytic use, unspecified with intoxication, uncomplicated'
265. 'Alcohol-induced mood disorder'
266. 'Opioid use, unspecified with opioid-induced psychotic disorder with hallucinations'
267. 'Cannabis use, unspecified'
268. 'Other psychoactive substance use, unspecified with psychoactive substance-induced persisting dementia'
269. 'Hallucinogen use, unspecified with hallucinogen-induced psychotic disorder with delusions'
270. 'Cocaine use, unspecified with cocaine-induced psychotic disorder with delusions'

271. 'Sedative, hypnotic or anxiolytic use, unspecified with sedative, hypnotic or anxiolytic-induced psychotic disorder with hallucinations'
272. 'Opioid abuse with opioid-induced psychotic disorder with hallucinations'
273. 'Inhalant use, unspecified, uncomplicated'
274. 'Alcohol-induced psychotic disorder with delusions'
275. 'Other psychoactive substance abuse with withdrawal, uncomplicated'
276. 'Cocaine-induced mood disorder'
277. 'Opioid-induced mood disorder'
278. 'Alcohol use complicating pregnancy, third trimester'
279. 'Hallucinogen dependence with other hallucinogen-induced disorder'
280. 'Anxiety disorder caused by stimulant'
281. 'Alcohol dependence with withdrawal'
282. 'Inhalant abuse with unspecified inhalant-induced disorder'
283. 'Cannabis use, unspecified with psychotic disorder with delusions'
284. 'No matching concept'
285. 'Sedative, hypnotic or anxiolytic abuse with withdrawal, unspecified'
286. 'Inhalant use, unspecified with inhalant-induced persisting dementia'
287. 'Hallucinogen abuse with intoxication with delirium'
288. 'Hallucinogen use, unspecified with hallucinogen persisting perception disorder (flashbacks)'
289. 'Sedative, hypnotic or anxiolytic abuse with withdrawal, uncomplicated'
290. 'Hallucinogen use, unspecified with hallucinogen-induced anxiety disorder'
291. 'Nicotine dependence, other tobacco product, with withdrawal'
292. 'Alcohol hallucinosis'
293. 'Inhalant use, unspecified with inhalant-induced mood disorder'
294. 'Hallucinogen abuse with hallucinogen-induced mood disorder'
295. 'Unspecified drug dependence, unspecified'
296. 'Hallucinogen abuse with hallucinogen-induced psychotic disorder with hallucinations'
297. 'Cannabis abuse with psychotic disorder with delusions'
298. 'Cocaine-induced psychotic disorder with hallucinations'
299. 'Alcohol dependence syndrome'
300. 'Insomnia caused by alcohol'
301. 'Cocaine delirium'
302. 'Mood disorder caused by stimulant'
303. 'Cocaine-induced organic mental disorder'
304. 'Cocaine use, unspecified with intoxication'
305. 'Alcohol induced disorder co-occurrent and due to alcohol dependence'
306. 'Opioid-induced mood disorder due to opioid abuse'
307. 'Other psychoactive substance dependence with intoxication delirium'
308. 'Alcohol abuse, with withdrawal'
309. 'Alcohol amnestic disorder'
310. 'Psychotic disorder caused by cocaine'
311. 'Cocaine intoxication'
312. 'Opioid use, unspecified with opioid-induced psychotic disorder'
313. 'Psychoactive substance-induced organic delusional disorder'
314. 'Other psychoactive substance dependence with withdrawal with perceptual disturbance'
315. 'Alcohol dependence with intoxication'
316. 'Cocaine delusional disorder'
317. 'Alcohol use, unspecified with withdrawal delirium'
318. 'Dementia associated with alcoholism'
319. 'Episodic chronic alcoholism'

320. 'Psychotic disorder caused by stimulant'

Schizophrenia source concept names

1. 'Residual schizophrenia'
2. 'Schizophrenia'
3. 'Other specified types of schizophrenia, in remission'
4. NA
5. 'Undifferentiated schizophrenia'
6. 'Other schizophrenia'
7. 'Paranoid type schizophrenia, chronic'
8. 'Unspecified schizophrenia, subchronic with acute exacerbation'
9. 'Disorganized schizophrenia'
10. 'Paranoid type schizophrenia, chronic with acute exacerbation'
11. 'Schizophrenic disorders, residual type, chronic'
12. 'Paranoid type schizophrenia, subchronic'
13. 'Other specified types of schizophrenia, unspecified'
14. 'Schizophrenic disorders, residual type, unspecified'
15. 'Unspecified schizophrenia, in remission'
16. 'Paranoid schizophrenia'
17. 'Schizophrenic disorders, residual type, chronic with acute exacerbation'
18. 'Simple type schizophrenia, chronic'
19. 'Paranoid type schizophrenia, unspecified'
20. 'Unspecified schizophrenia, unspecified'
21. 'Unspecified schizophrenia, chronic'
22. 'Schizophrenia, unspecified'
23. 'Simple type schizophrenia, unspecified'
24. 'Catatonic type schizophrenia'
25. 'Disorganized type schizophrenia, chronic'
26. 'Unspecified schizophrenia'
27. 'Unspecified schizophrenia, subchronic'
28. 'Schizophrenic disorders, residual type, subchronic with acute exacerbation'
29. 'Disorganized type schizophrenia, unspecified'
30. 'Other specified types of schizophrenia, chronic'
31. 'Paranoid type schizophrenia, in remission'
32. 'Unspecified schizophrenia, chronic with acute exacerbation'
33. 'Catatonic schizophrenia'
34. 'Catatonic type schizophrenia, unspecified'
35. 'Chronic residual schizophrenia'
36. 'Simple type schizophrenia, subchronic'
37. 'Latent schizophrenia, subchronic with acute exacerbation'
38. 'Other specified types of schizophrenia, chronic with acute exacerbation'
39. 'Paranoid type schizophrenia, subchronic with acute exacerbation'
40. 'Simple type schizophrenia, chronic with acute exacerbation'
41. 'Paranoid type schizophrenia'
42. 'Disorganized type schizophrenia, subchronic with acute exacerbation'
43. 'Simple type schizophrenia, subchronic with acute exacerbation'
44. 'Lethal catatonia'
45. 'Other specified types of schizophrenia, subchronic'
46. 'Catatonic type schizophrenia, in remission'
47. 'Other specified types of schizophrenia, subchronic with acute exacerbation'

48. 'Paraphrenia'
49. 'Simple schizophrenia'
50. 'Disorganized type schizophrenia'
51. 'Catatonic type schizophrenia, subchronic with acute exacerbation'
52. 'Schizophrenic disorders, residual type, in remission'
53. 'Other specified types of schizophrenia'
54. 'Disorganized type schizophrenia, subchronic'
55. 'Simple type schizophrenia'
56. 'Disorganized type schizophrenia, chronic with acute exacerbation'
57. 'Simple type schizophrenia, in remission'
58. 'No matching concept'
59. 'Catatonic type schizophrenia, chronic'
60. 'Chronic schizophrenia'
61. 'Schizophrenic disorders'
62. 'Schizophrenia in remission'
63. 'Subchronic undifferentiated schizophrenia'
64. 'Catatonic type schizophrenia, chronic with acute exacerbation'
65. 'Chronic paranoid schizophrenia'
66. 'Acute exacerbation of chronic paranoid schizophrenia'
